# Visual place recognition with panoramic images using hybrid neural network models

**DOI:** 10.1038/s41598-025-34473-7

**Published:** 2026-01-08

**Authors:** Lars Offermann

**Affiliations:** Faculty of Technology, Bielefeld University, Bielefeld, 33615 Germany

**Keywords:** Engineering, Mathematics and computing

## Abstract

A mobile robot can localize itself in a mapped area by finding a recorded image of a visited place that is most similar to the current view, a technique known as Visual Place Recognition (VPR). We focus on VPR with panoramic images in indoor environments and on direct VPR methods in contrast to feature-based methods. In this context, a key challenge of VPR are appearance changes in the environment, e.g. due to variations in illumination, camera tilt, and rearrangement of objects. To improve the quality in these situations, we propose a novel combination of convolutional neural networks (CNNs) for image preprocessing with two algorithmic solutions for VPR, the Visual Compass and MinWarping. Here, the CNN is fused with the algorithmic VPR method such that the training of the neural network includes backpropagation through both parts, which we refer to as a hybrid model. We show that the hybrid Visual Compass substantially improves tilt tolerance, resulting in a versatile model, while hybrid MinWarping is especially robust against illumination changes and object rearrangement. As an adjacent application to VPR, the hybrid MinWarping algorithm can also be used to estimate the relative pose of the query with respect to a previous image. We analyze how the network solution to image processing changes fundamentally to satisfy the unique requirements of each application. We also show that the VPR hybrid models compare favorably for upright images with a competing solution based on sparse local features.

## Introduction

In mobile robotics, recognizing a place by its appearance means localizing an agent in the world; this task is known as visual place recognition (VPR). As a prerequisite, the agent must capture images of its surroundings during an exploration phase, which results in an image-based map. Localization in this map is then carried out as a similarity search of the agent’s current camera view with respect to the images in the map. In this context, the current view is referred to as a *query*. VPR becomes increasingly difficult the more the view of a place has changed since the exploration, e.g. due to variations of illumination, changes of the viewpoint, or movement of objects.

Panoramic images are especially well suited for VPR^1^ due to their full surround view, which helps to mitigate the dependency on specific viewpoints. Throughout this work, we investigate algorithms specifically designed for this image type. To this end, we use images that are captured using a single, upward facing camera with an ultra wide angle fisheye lens - a simple and cost-effective solution^2^. Images captured with this setup offer a full panoramic view along the horizontal axis, but the field of view along the vertical axis is limited. Therefore, viewpoint changes due to camera tilt still pose a challenge.

**Fig. 1:**
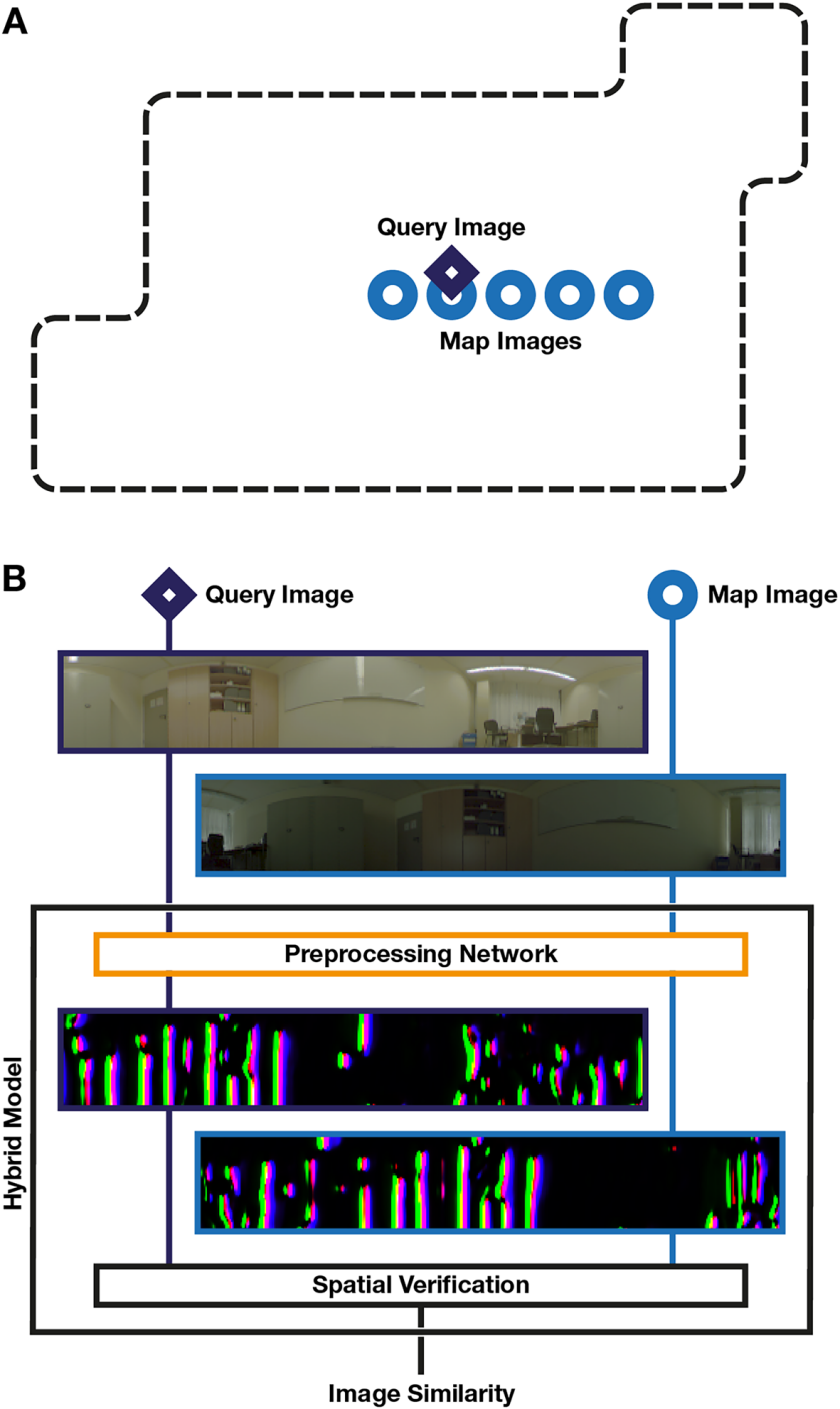
Overview of Visual Place Recognition (VPR) with the proposed hybrid models. (**A**) In the past, an agent has captured *map images* (light blue circles) of the *environment* (dashed line). Now, the agent localizes itself in the map using its current view, which it uses to *query* (dark blue diamond) the map. (**B**) Schematic of a hybrid model to compare an image pair of query and map image. Adding the preprocessing network (orange) is our contribution. A threshold on the final image similarity is used to determine if images were taken at the same place.

In our previous work^3^, we investigated an adjacent application to VPR, the relative pose estimation (RPE) of the current camera view with respect to a previous image. For RPE, learning how to preprocess images for a selected algorithmic solution substantially improves the tolerance to appearance changes in the environment. This cooperation between the network and the RPE algorithm can be implemented effectively by embedding the algorithmic solution into the neural network as integral part during training and estimation. We refer to this compound architecture as a *hybrid model*. In this work, we apply this concept to VPR and compare preprocessing solutions between both applications. An overview of the application of VPR to robot localization and the role of the hybrid model is presented in Fig. 1.

We follow the state of the art in VPR as described by Masone and Caputo^4^ and view VPR as a system of separate phases. The first phase involves the creation of a compact, yet distinguishable representation for every image in the map: a global image descriptor. When localizing the robot, the same method is used to create a descriptor for the query. Based on this, a fixed number of the most similar descriptors is retrieved from the map. Subsequently, we refer to the combination of global descriptor extraction and similarity search as *image retrieval*. For this step, we rely on the CosPlace neural network^5^ with minor modifications to better support panoramic images. The results of this step are then refined in a second phase using methods that are more discriminative but also more computationally demanding.

We focus on *spatial verification* as one such approach to refinement. Spatial verification refers to using a geometric model that relates the camera movement in robot coordinates to the landmark movement in image coordinates. The image similarity between a query and a map image is then derived from the agreement of the observed landmark movement to the geometric model.

We propose two hybrid models built upon spatial verification methods that are designed to be used with panoramic images, the *Visual Compass*^6^ and *MinWarping*^7^. These models are well suited for the integration into CNN-based hybrid models, because, like CNNs, they directly operate on the dense pixel information of input images.

Both methods calculate an image distance for VPR, but the Visual Compass is able to compensate for 2D rotations along the horizontal axis while MinWarping accounts for both the 2D rotation and a 2D direction of translation between views. We show that hybrid MinWarping is especially suited for upright images and variations in illumination, while the hybrid Visual Compass substantially improves robustness against camera tilt but only provides limited illumination tolerance. Then, we compare hybrid MinWarping models for RPE and VPR and find that their visual processing requirements differ fundamentally, which is revealed by the different preprocessing solutions found by the networks.

Finally, we compare the proposed hybrid models to spatial verification based on sparse local features and a suitable geometric model as described in multiple works^4,8–11^. Here, we show that the popular inlier-based scoring method is not suitable for the investigated datasets and instead propose an alignment-based scoring. We observe that the VPR quality of spatial verification with sparse local features is advantageous for situations with tilted viewpoints and it is also competitive for movement in the plane and coarse grid spacings. For any grid spacing and planar movement, the proposed hybrid models offer the best precision in our testing.

Experiments use the grid-based image datasets of our previous work^3^, which we extend with a new dataset of office and laboratory scenes that captures object movement and camera tilt.

The remainder of this paper starts with an overview of related work in the “Related work” section. Then, we outline the capturing, processing, and use of datasets in the “Datasets” section. The section “Methods” describes our experimental setup, the used performance metrics, the setup and modification of CosPlace, and the design of the hybrid models. Following this, we configure the loss function for training hybrid models using validation data and assess the VPR quality for CosPlace and hybrid models (Sect. “Results”). A discussion of our findings can be found in the “Discussion” section. The final section “Conclusion” contains the conclusion and outlook.

## Related work

In the following, we outline image retrieval and spatial verification for refinement as separate phases of VPR.

### Image retrieval

Image retrieval methods enable fast search even in large maps. Specifically, they make the comparison of spatial verification methods tractable for the datasets used in this work. To realize this, compact global image descriptors are created for query and map images that retain discriminability between different places. Global descriptors for image retrieval can be aggregated from sparse local feature descriptors (e.g. Bag of Words^12^, VLAD^13^, Fisher Vectors^14^), combined from dense local features (from Convolutional Neural Networks (CNNs)^5,15^ or Vision Transformers^16–18^) or created directly by analysing the distribution of local or global frequencies in the input image (e.g. local: GIST^19^, HoG^20^; global: Fourier signatures^21^). For an overview of recent methods, we refer to the work of Masone and Caputo^4^.

In this work, we use the recent CNN-based image retrieval method CosPlace^5^, as it is used as a competitive baseline in recent works on VPR^16,17,22,23^ and it works well with panoramic images after minor modifications (see "Image retrieval with CosPlace").

We use image retrieval to search for panoramic images in a map of the same image type. This reflects an application in which an agent captures snapshots in an exploration phase, then later localizes itself within this map. Therefore, the camera setup and image type are expected to be identical. This contrasts approaches that use *perspective* images (e.g. from a smartphone camera) for localization in a map of panoramic images. Solutions to VPR for these applications are discussed in the works of Shi et al.^24^ and Gard et al.^25^.

### Refinement with spatial verification

Spatial verification for VPR means finding a hypothesis that best explains the movement of corresponding local features between a query and a map image. The agreement of feature matches to the hypothesis leads to a new estimate of the image similarity, replacing the solution of the image retrieval step. We focus on spatial verification methods that build upon geometric models to explain feature movement, with alternatives including heuristics like the rapid spatial scoring^9^ and machine-learning-based scoring^8^. Spatial verification is commonly implemented on the basis of sparse local image features^9,26^ and a selected geometric model, e.g. affine transformations in the work of Noh et al.^26^ or 2D homographies in the work of Hausler et al.^9^.

These solutions assume a perspective projection model and a flat imaging surface, but this is violated by spherical camera model and the circular panoramic images used in this work (see “Datasets”). To establish a competitive baseline for the proposed hybrid models, we instead follow Gálvez-López and Tardos^27^ and estimate a relative camera pose using epipolar geometry^28^. Fitting the geometric model for matches of sparse local features is typically realized with RANSAC^29^, which includes outlier rejection, with the image similarity then being the number of inliers^4,8,9^. In the section “Results”, we show that the inlier count is an insufficient similarity score for the tested datasets, and instead propose a distance metric based on aligning the camera frames in the section "Spatial verification with sparse local features".

As an alternative to sparse local features, we build upon dense pixel-wise information for spatial verification that are specifically designed for panoramic images. Suitable models were suggested by several works^6,30,31^: The *Visual Compass* assumes a planar camera motion between panoramic query and map images and computes a similarity score based on the minimum pixel difference of all possible rotations. The *MinWarping* algorithm^7^ also operates on a pair of panoramic query and map images but uses a geometric model with planar motion assumption to estimate a relative camera pose. In both cases, the score of the best fitting model is used as similarity score for VPR^31^.

## Datasets

**Fig. 2:**
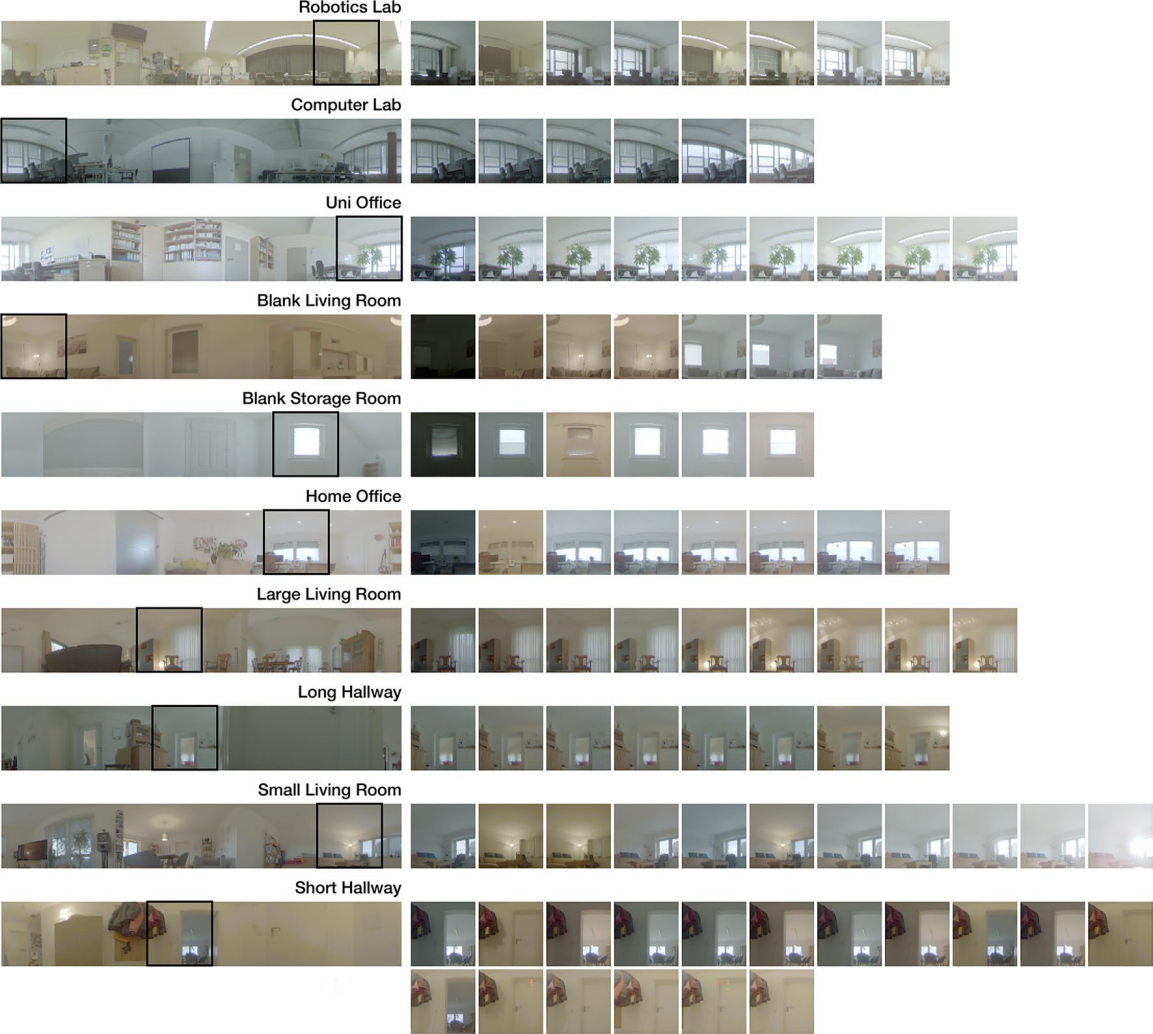
Overview of the panoramic image datasets used for training and validation. The left column shows one example image for each setting, the right columns represent illumination variants for the marked section in the example image. This figure has been presented in our previous work^3^ alongside details on the dataset recording and processing.

Throughout this work, we use panoramic color images in an equirectangular format, which are captured with an upward-facing fisheye camera. We record high dynamic range (HDR) data to improve visibility in especially dark and bright areas. Examples are shown in Fig. 2, among others.

For systematic evaluation, we use datasets with images taken at the nodes of regular grids. All images are annotated with a metric pose relative to the grid origin. Each pose corresponds to the 2D position in the x-y plane of a right-handed coordinate system and the 2D tilt as rotation angles around the x- and y-axes. The camera height and the rotation around the z-axis are kept constant. Within every capture area, the same number of images are taken at every grid node, with each image reflecting a predetermined appearance change (e.g. illumination). In this case, capture areas are open spaces of home, office, or lab environments. We refer to a specific capture area as *setting* while a *variant* is an instance of appearance change.

We partition image datasets into three disjoint parts: one part for the automatic fitting of model parameters (*training*), a second part for the manual optimization of parameters (*validation*), and a third part for an independent evaluation (*test*).

For training and validation, we use the square-grid-based datasets with varying illumination introduced in our previous work^3^; see Fig. 2 for an overview. For training, we use all settings except Robotics Lab, which is retained for validation. These datasets capture changes of illumination but no rearranging of objects or camera tilt. Illumination changes include natural sources like daytime and weather, the use of shutters, and variations of artificial light sources.

To complement the validation dataset and enable testing with additional appearance changes, we recorded three new settings captured in offices and laboratories in Bielefeld University: Gantry Lab, Computer Lab II, and Robotics Lab II. Compared to their versions introduced above, Computer Lab II is captured in a different area of the room and with changed furniture. For Robotics Lab II, the capture area has been moved closer to surrounding objects. Additionally, the new datasets not only capture illumination changes but also changing object arrangements and camera tilt. To support evaluation of tilted images and moved objects in the validation dataset, we select Robotics Lab II for this partition and complement it with the data of our original Robotics Lab setting. Computer Lab II and Gantry Lab are retained for testing. The partitioning of data into training, validation, and test splits is summarized in Table 1.

**Table 1:** Summary of data partitioning by settings.

Data Partition	Settings
Training	Computer Lab, Uni Office, Blank Living Room, Blank Storage Room, Home Office, Large Living Room, Long Hallway, Small Living Room, Short Hallway
Validation	Robotics Lab, Robotics Lab II
Test	Gantry Lab, Computer Lab II

Images for the three new settings are captured at nodes of an equilateral triangle grid with an edge length of 5 cm, resulting in 2170 images in an area of 1.75 m by 2.7 m. The triangle grid is laid out in 62 rows of 35 images. Images within a row are aligned with the x-axis and every second row is shifted by 2.5 cm. Note that the spacing between images must be viewed in relation to the scale of the environment due to the geometric relation between any observed landmark and the capture locations of a particular pair of dataset and query image. This is because the image difference induced by the movement within the image pair becomes easier to detect as the ratio between the distance of capture locations and the distance to the landmark increases. We mitigate the issue by choosing typical home and office environments to limit the variation in scale between settings; see Sect. S1 in the supplementary information for maps of the environments.

**Fig. 3:**
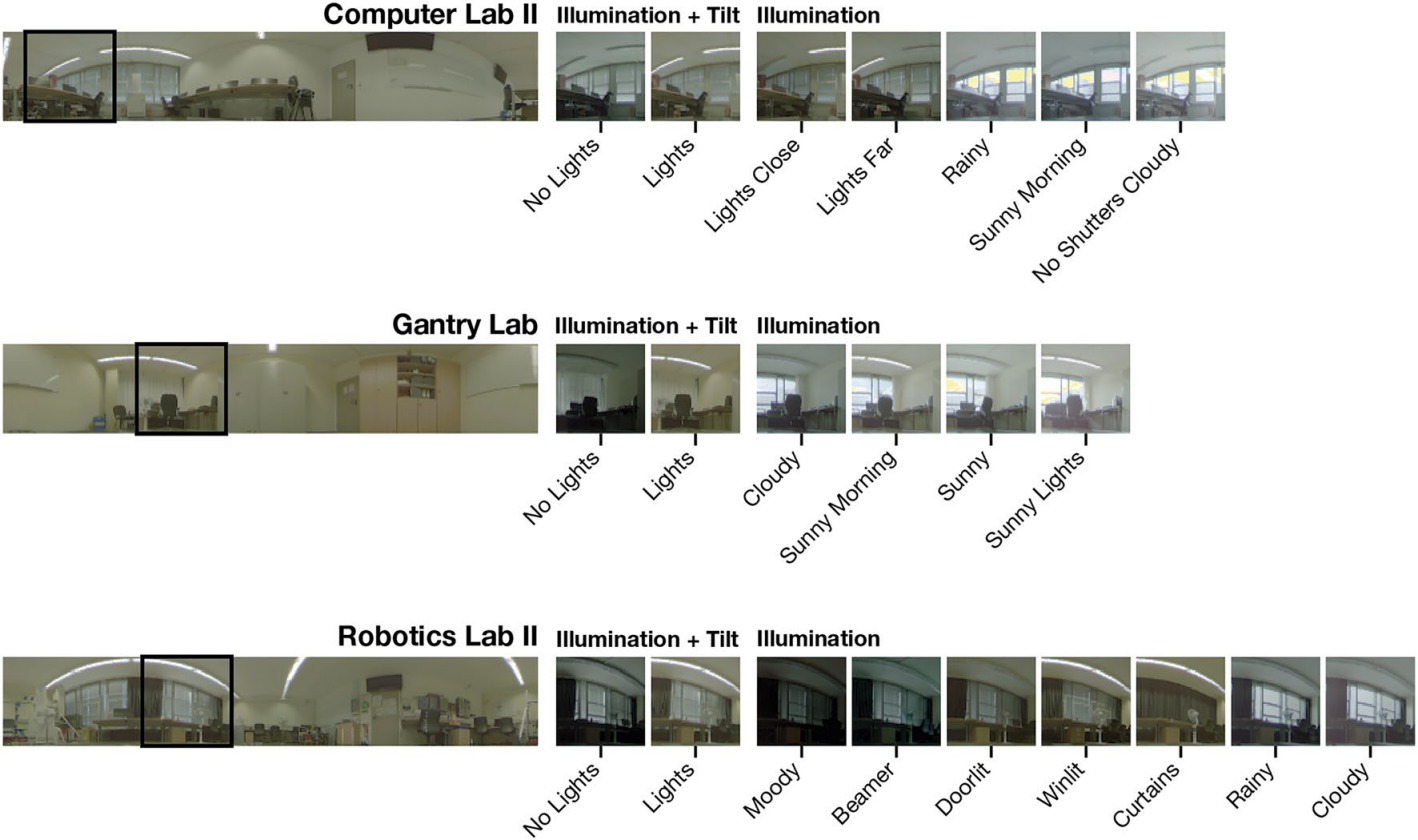
New dataset: Overview of the recorded variants per setting used for the validation and test partitions. On the left side, we show a reference image for each of the settings Computer Lab II, Gantry Lab, and Robotics Lab II. For the marked section in the reference image, we display variants with illumination changes and camera tilt on the right.

Illumination variants without tilt include natural lighting. As the capturing of a single variant takes approximately 1:45 h, these variants may exhibit slight changes of illumination between capture positions. For variants with camera tilt, we purposefully limit the influence of natural lighting to reduce illumination variance. An overview of variants for each setting is shown in Fig. 3.

**Fig. 4:**
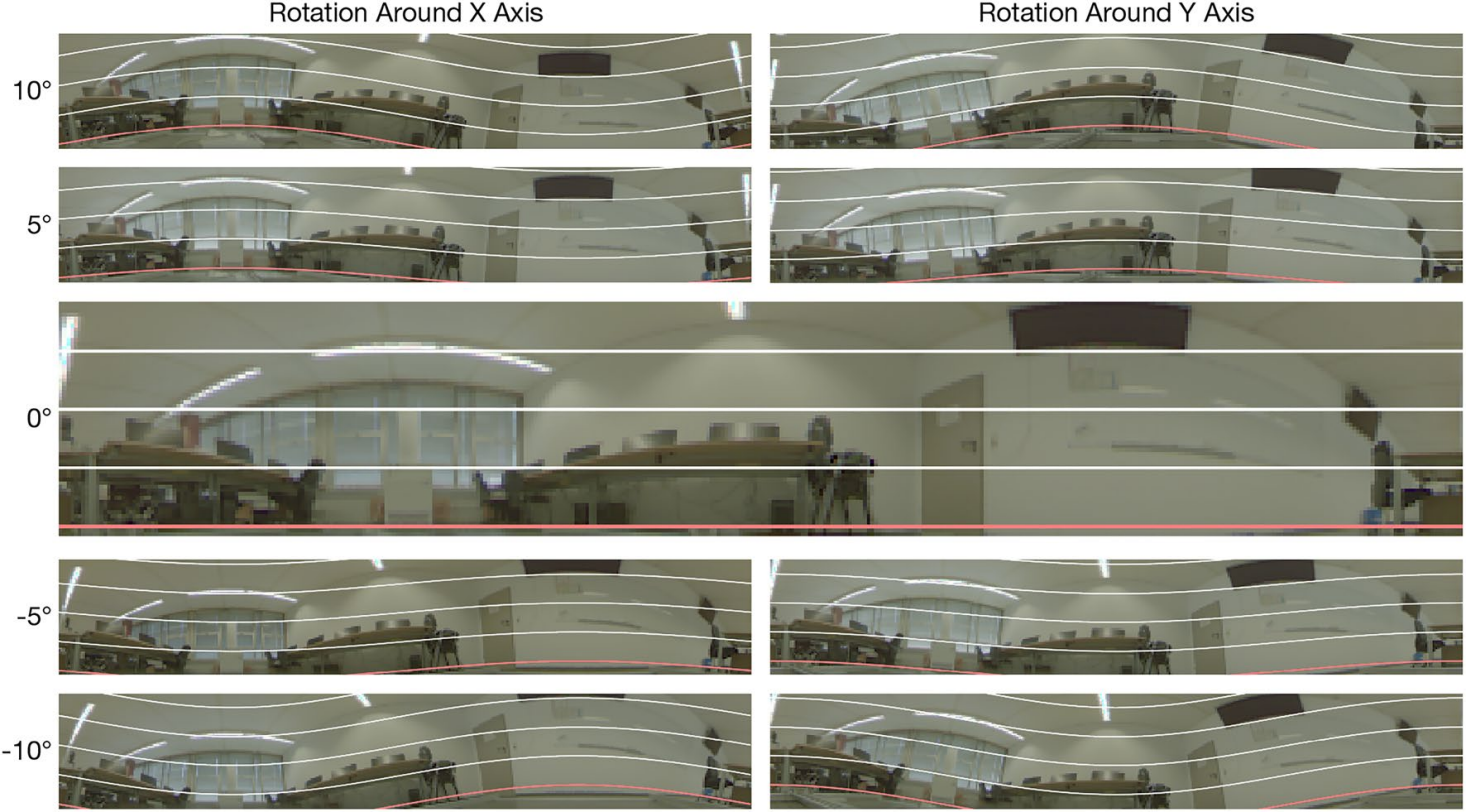
Overview of tilt variants using Computer Lab II. The images are taken near the center of the capture area at grid index (17, 31). The camera was either rotated around the x- or y-axis in 5$^{\circ }$ steps with respect to a right-handed coordinate system. Overlayed lines show the latitudes of a spherical coordinate system in 10$^{\circ }$ steps, the red line marks the horizon.

Variants with camera tilt can be used to simulate uneven and changing ground. To this end, we complement the upright illumination variants *Lights* and *No Lights* (see Fig. 3) for each setting with 8 tilted images at each grid node. The camera is rotated either around the x- or y-axis of the coordinate system by $\pm 5^{\circ }$ and $\pm 10^{\circ }$. Recording tilted images for two illumination variants allows us to test simultaneous changes of tilt and illumination. The effect of tilt angles on the camera viewpoint is shown in Fig. 4.

**Fig. 5:**
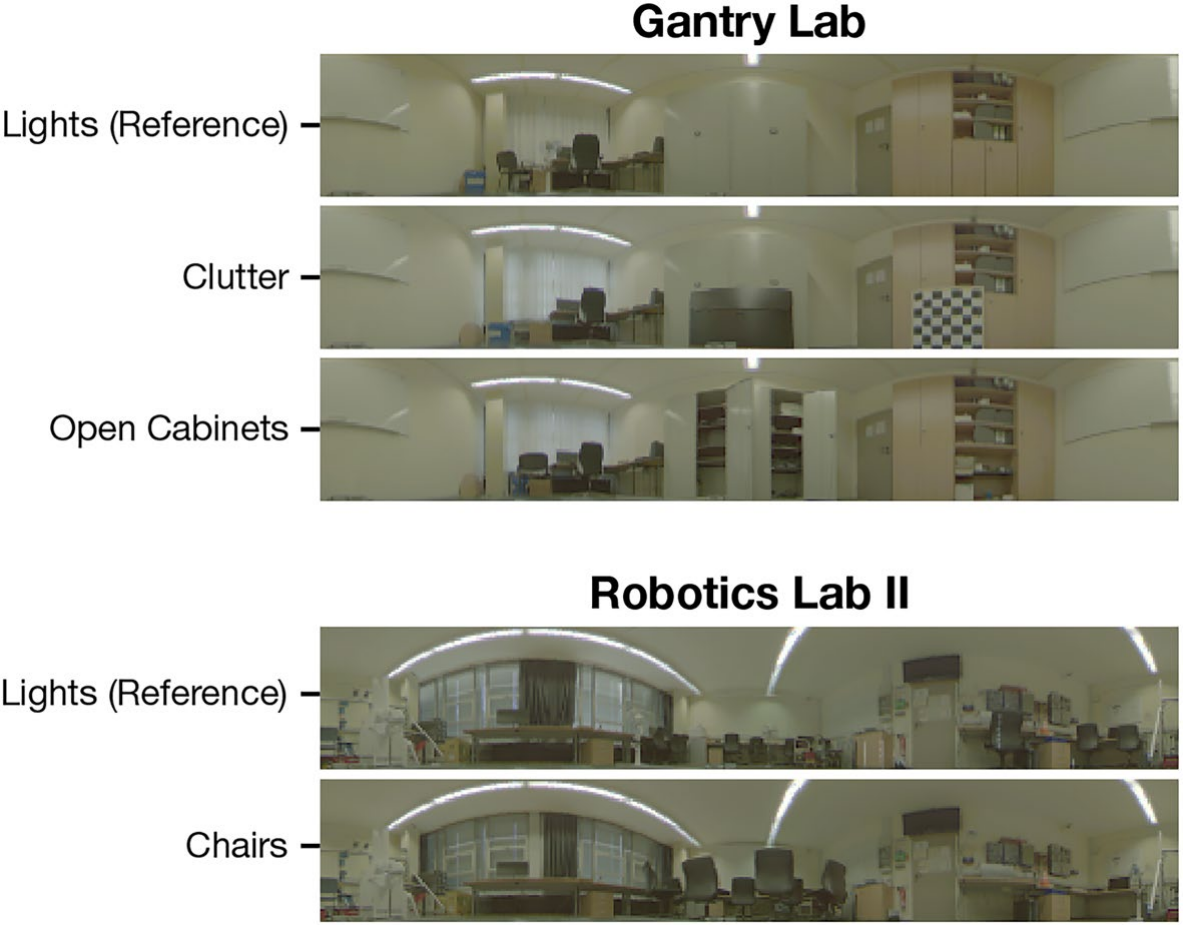
Overview of variants with rearranged objects for the settings Gantry Lab and Robotics Lab II. The first variant, “Lights” is included for reference.

Variants with rearranged objects represent an instance of change in the scenery due to movement of chairs, additional objects, and opening or closing of cabinets. An overview of these variants is shown in Fig. 5.

A detailed description of settings and variants is provided in Table 2.

**Table 2:** Overview of dataset with descriptions of settings.

Setting	Description	Illumination Variants *with Tilt*	Illumination Variants *without Tilt*	Variants with Rearranged Objects
Computer Lab II	Seminar room with 3 table groups. Each table group offers a desktop computer setup per seat. There are two entrances on one side of the room and windows on the opposite side. Next to each entrance is a free space surrounded by furniture. In contrast to the Computer Lab setting from our previous work^3^, the furniture and equipment have changed and the opposite free space is used as a capture area. The density of objects is medium and the distribution is even.	Both variants include reduced illumination through the windows using shutters. *Lights:* all ceiling lights are switched on *No Lights:* all ceiling lights are switched off	*Lights Close:* light from the windows is reduced using shutters, ceiling lights above the capture area are on. *Lights Far:* illumination from the windows is reduced using shutters, ceiling lights above the free space opposite the capture area are on. *Rainy:* clear view through the windows, rainy weather. *Sunny Morning:* clear view through the window, sunny weather. *No Shutters Cloudy:* clear view through the window, cloudy weather.	None
Gantry Lab	Office with a single entrance, two metal cabinets, and a cabinet/bookshelf combination. Opposite of the wall there is a desk with a computer setup and a row of windows. The density of objects is medium on three sides of the capture area; the fourth side is taken up by a blank wall with a whiteboard.	Both variants include reduced illumination through the windows using shutters and curtains. *Lights:* both ceiling lights are switched on *No Lights:* both ceiling lights are switched off	All variants in this category feature a clear view through the window. *Cloudy:* cloudy sky *Sunny Morning Lights:* sunny weather in the morning and ceiling lights *Sunny:* sunny weather mid-day *Sunny Lights:* mid-day sunny weather and ceiling lights	*Open Cabinets:* rearranged objects at desk area, steel and wood cabinets are partly opened *Clutter:* rearranged objects at desk area, additional black box and calibration pattern
Robotics Lab II	Lab environment with tables and experimental equipment surrounding a free space in the center. There is a single entrance and windows on the opposite side. Illumination from the windows is controlled with shutters and an opaque curtain. There is a projection screen that can be unrolled on free wall. The density of objects is high and the distribution is even.	Both variants include reduced illumination through the windows using shutters. *Lights:* both ceiling lights are switched on *No Lights:* both ceiling lights are switched off	*Moody:* shutters and two-point lights *Beamer:* shutters, white image shown on projection screen *Winlit:* shutters and window-side ceiling lights *Doorlit:* shutters and doorside-ceiling lights *Curtains:* curtains and both ceiling lights *Rainy:* clear view through the windows, rainy weather *Cloudy:* clear view through the windows, lightly cloudy weather	*Chairs:* rows of chairs in the free space, projection screen

### Dataset collection

**Fig. 6:**
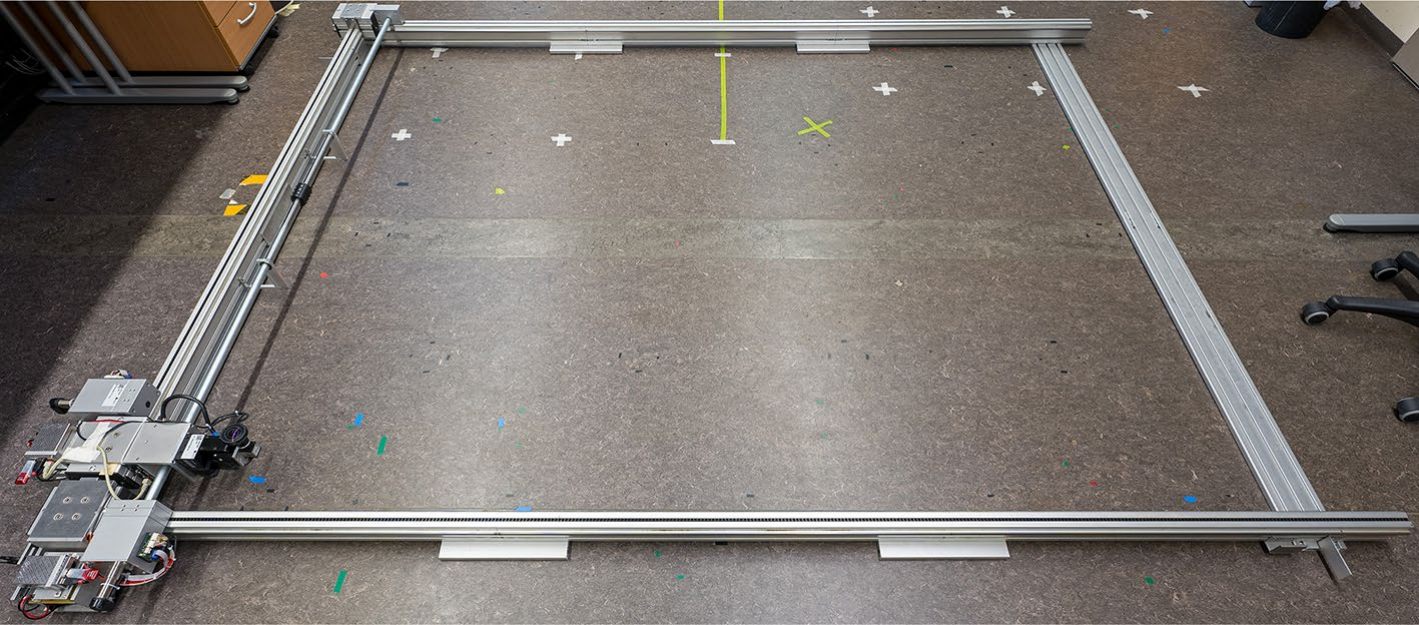
Overview of the area gantry setup as it is used for recording the setting Computer Lab II.

**Fig. 7:**
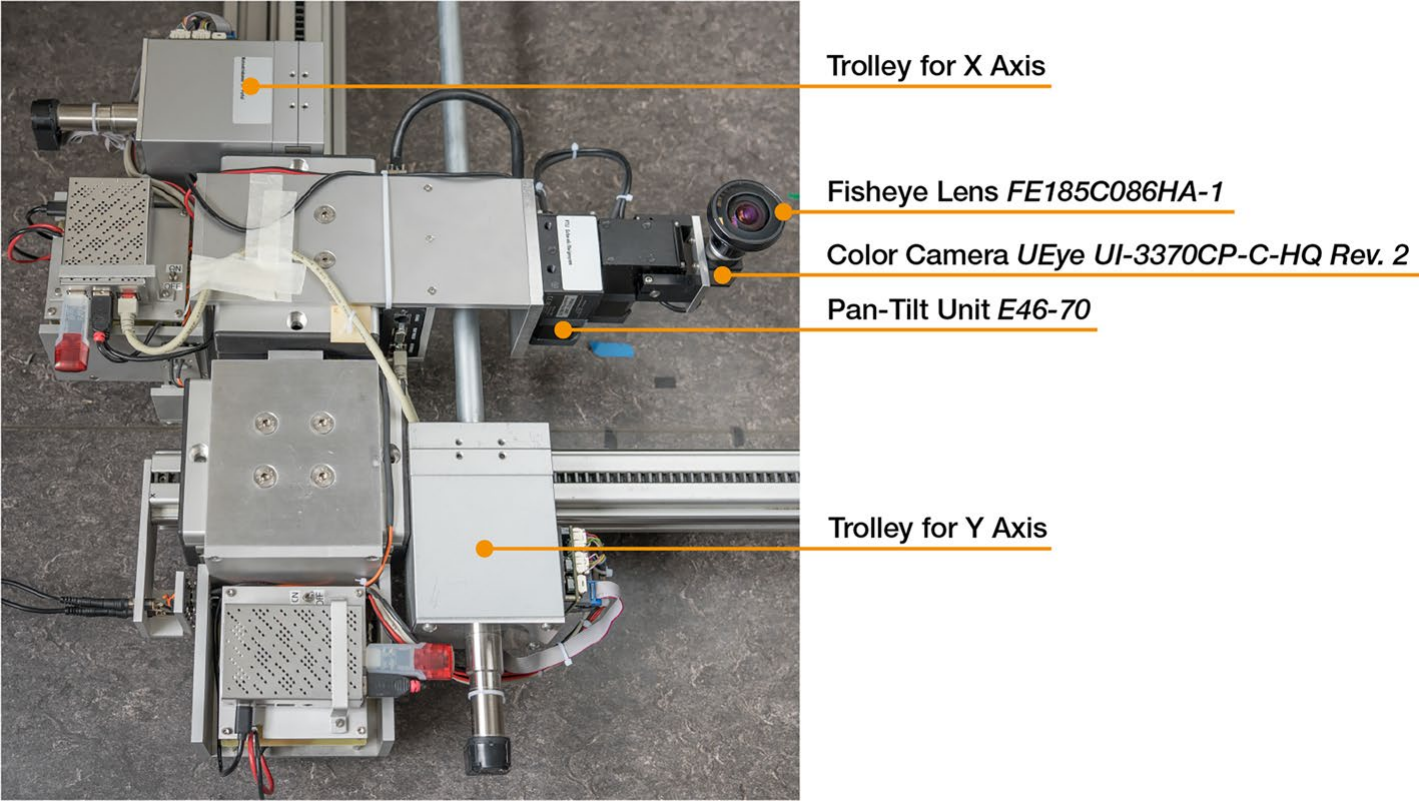
Detail view of moving parts of the area gantry. Manufacturer logos and inventory numbers were removed using image processing.

The new dataset is recorded using an area gantry with two horizontal axes that allow planar motion of the trolleys parallel to the ground. The trolley for the x-axis is equipped with a Flir E46-70 Pan-Tilt Unit (PTU) with a resolution of 0.003$^{\circ }$ on the pan and tilt axes. The PTU is mounted as described by Berganski et al.^32^ such that a pan or tilt becomes a rotation around the x- or y-axis of the coordinate system of the gantry and there is no rotation around the z-axis. The PTU is moving an IDS UEye UI-3370CP-C-HQ Rev. 2 color camera with a Fujifilm FE185C086HA-1 circular fisheye lens. The opening angle of the lens amounts to 185$^{\circ }$. We keep the aperture of lens fixed to F1.8. An overview of the area gantry setup is shown in Fig. 6 and Fig. 7.

We use a grid spacing of 5 cm and ensure that the precision and repeatability of the positional control of the gantry is sufficient for this grid resolution by repeatedly moving the camera to the same positions in an environment with constant illumination, capturing images, and analyzing the image distances. Details on the procedure can be found in Sect. S2 in the supplementary information (Fig. 7).

To capture the color HDR images, we first set a fixed gain of (50,39,65) for the camera sensor, corresponding to red, green, and blue channels with respect to an 8-bit range. At each grid node, we then take 5 images using the double exposure mode of the camera, successively halving the exposure time from 51.2 ms to 0.1 ms. This results in 10 images of the size 2048 $\times$ 1024 px. Each image in the exposure series is low-pass filtered using a third-order Butterworth filter with zero shift and a relative width of 0.2.

Assuming a spherical camera model, we map every image it to a 384 $\times$ 99 px equirectangular format using the intrinsic camera parameters determined by calibration using the toolboxes of Scaramuzza et al.^33^ with improvements by Urban et al.^34^. Then, we fuse the remapped images into a single HDR image with the method by Robertson et al.^35^.

Pixels that are overexposed in every image of the series cause discoloration artifacts. We mitigate this problem by setting the HDR color value of a pixel with a mean value larger than 247 (8-bit range) in the exposure series to the maximum value of this color within all HDR images in the dataset. HDR images are cropped vertically from 99 px to 64 px by cutting off the topmost pixels to remove areas with excessive distortion. Then, images are converted to an 8-bit range by applying the natural logarithm, normalizing and scaling to the range [0,255], then discretizing to integers. We simulate different forward directions of the robot’s viewpoint by shifting the panoramic image along the horizontal axis by uniformly drawn integers.

These camera hardware and preprocessing steps follow our previous work^3^ to ensure compatibility of the image formats between datasets.

## Methods

In the following, we report on the experimental setup and the metrics used to measure place recognition quality. Then, we introduce CosPlace, the hybrid Visual Compass, and hybrid MinWarping. Lastly, we outline our implementation of VPR with sparse local features, including scoring with inliers and our proposed alignment-based metric.

### Experimental setup

The following experiments require the training, validation, and test partitions described in the “Datasets” section.

The usage of the training partition is particular to the specific method and is described individually. For validation and test datasets, we select subsets of variant pairs. Each subset corresponds to a specific type of appearance change, e.g. illumination or tilt, and is referred to as an *appearance subset*. During evaluation, the first variant of any pair is used as a map, while any image of the second variant is used as query.

For Computer Lab II, Gantry Lab, and Robotics Lab II, we devise 7 appearance subsets. The first one contains variant pairs with mixed illumination and no tilt. The next four appearance subsets represent 5$^{\circ }$ or 10$^{\circ }$ tilt, each with constant or mixed illumination. Here, only one variant is tilted by 5$^{\circ }$ or 10$^{\circ }$, while the other one is upright. The last two appearance subsets contain pairs with rearranged objects and constant or mixed illumination. Pairs that contain the same variant twice correspond to no environmental changes between query and map images and are excluded from the evaluation.

For Robotics Lab, we create a single appearance subset for mixed illumination. This set and all appearance subsets of Robotics Lab II comprise the validation partition. The test partition contains all appearance subsets of Computer Lab II and Gantry Lab.

To assess the quality of a VPR method for one such subset, we process variant pairs independently and select images from one variant as the map and images from the other variant as query. This corresponds to an application in which the robot has recorded the map in a single run and is locating itself after some time has passed. The new settings Computer Lab II, Gantry Lab, and Robotics Lab II are evaluated with multiple subsampling steps that are applied simultaneously to the map and query variant, corresponding to a grid spacing of 10 cm (2x subsampling), 20 cm (4x), and 40 cm (8x). Because the training partition only offers grids with 20 cm spacing, all methods in this work are optimized for this setup and the finer grid is included as an additional test. The evaluation of the native grid spacing of 5 cm is left for future work. Any subsampling is applied such that every row of the triangle grid contains an identical number of images, possibly removing the image column with the x-coordinate farthest away from the origin.

We follow the common approach^4,36,37^ and measure the quality of a VPR method by applying a threshold to image similarities to differentiate between same and different places. For this purpose, we label image pairs as taken at the same position if their x and y-coordinates with respect to the grid are identical. These images comprise the set of positive examples $P_{i,j}$ for the $i$-th setting and the $j$-th variant pair.

### Metric for image retrieval: Recall@K

In the first phase of VPR, we are using CosPlace to retrieve the $K$ images from the map that are most similar to a query. In the second phase, spatial verification is only applied to this selection. Therefore, positive examples that evade the retrieval step cannot be found. In this context, retrieving all positives for a given query is the defining quality of the image retrieval step. This is measured using the recall@K $R_{K,i,j} = \frac{|\textit{TP}_{i,j}|}{|P_{i,j}|}$, with $\textit{TP}_{i,j}$ as the set of true positives and $P_{i,j}$ as the set of positives (see “Experimental setup”). The average recall across settings is computed via the average of the individual recall@K for the $N_i$ variant pairs of the $i$-th setting (“macro-averaging”^38^): $R_{K}=\frac{1}{M} \sum _{i=1}^M(\frac{1}{N_i}\sum _{j=1}^{N_i} R_{K,i,j})$.

### Metrics for spatial verification: the area under the precision-recall curve (PR AUC)

We are using spatial verification as a method to refine the result of image retrieval by re-computing the similarity for all retrieved candidates. A threshold then discriminates between images of the same and different places. Choosing the threshold is a tradeoff between the precision $P_{i,j} = \frac{|\textit{TP}_{i,j}|}{|\textit{TP}_{i,j}|+|\textit{FP}_{i,j}|}$ and the recall $R_{K,i,j}$, with $\textit{TP}_{i,j}$ and $R_{K,i,j}$ as defined in the section "Metric for image retrieval: Recall@K" and $\textit{FP}_{i,j}$ as the set of false positives for the $i$-th setting and the $j$-th variant pair. We test 100 equally spaced thresholds between the minimum and maximum of all similarities of variant pairs for a selected appearance subset (e.g., all similarity pairs with mixed illumination in all test settings). We assume that an image shows the same place if the similarity is larger or equal to the threshold. When evaluating all $M$ settings and $N$ variant pairs of an appearance subset, we aggregate the recall as $R_{K}=\frac{1}{M} \sum _{i=1}^M(\frac{1}{N_i}\sum _{j=1}^{N_i} R_{K,i,j})$ and the precision as $P=\frac{1}{M} \sum _{i=1}^M(\frac{1}{N_i}\sum _{j=1}^{N_i} P_{i,j})$. For thresholds that yield $R_{K,i,j} = 0$, we define $P_{i,j} = 1.0$.

Collecting the $P, R_K$ for all thresholds results in a precision-recall curve, a standard tool for evaluating the quality of VPR algorithms^37,39^. Note that the maximum achievable recall when applying spatial verification as a refinement step is determined by the method used for image retrieval, in this case, CosPlace.

The precision-recall curve is used to select a threshold for classification of images at runtime by choosing the acceptable tradeoff between recall and precision for a given VPR system and a target application. Additionally, the area under the precision-recall curve (PR AUC) allows for a general assessment of the quality of a VPR system^36^.

Because spatial verification after image retrieval is an optional refinement step, we are also assessing the PR AUC for the CosPlace as an additional baseline.

### Image retrieval with CosPlace

Since the maximum recall of the spatial verification method is determined by the image retrieval step, we are interested in configuring CosPlace to achieve the ideal VPR quality in the tested environments. To this end, we choose a suitable CNN architecture for feature extraction, adapt the network architecture to better support panoramic images by applying *circular padding*, and fine-tune the network weights using the training partition. In Section Recall with CosPlace, we show that circular padding and fine-tuning CosPlace with the selected CNN substantially improves the recall on the validation partition.

#### CosPlace architecture and circular padding

The CosPlace architecture builds upon a convolutional neural network pre-trained for image classification, e.g. VGG^40^ or ResNet^41^. The feature maps created by the last convolutional layer of the CNN are treated as a collection of local features, which get extracted at regular grid positions of the input image. These local features are combined using generalized mean pooling (GeM-Pooling^42^). The final image descriptor is then created from the pooled features using a single densely connected network layer.

We use the implementation of CosPlace^5^ provided by the authors (https://github.com/gmberton/CosPlace). Following their work, we tested VGG16, ResNet18, and ResNet50 as feature extraction networks. Since it offers the best recall@10 on upright validation data with 20 cm spacing, we select ResNet50 for all experiments.

Finally, we adapt the padding behavior of the CNN-layers of ResNet50 for the horizontal image axis from zero padding to circular padding, which repeats the opposite image edge. This method was suggested by multiple authors^43,44^ to prevent padding artifacts in panoramic images. Using circular padding, we observe substantially improved place recognition quality when the view is shifted along the horizontal direction.

#### Fine-tuning

Next, we describe the training procedure and the corresponding setup of our dataset to fine-tune the CosPlace weights. Then, we outline the selected training parameters.

The CosPlace architecture is trained for classification with the large margin cosine loss introduced by Wang et al.^45^. To this end, a separate densely connected layer is introduced that learns the affinity of a global feature descriptor to a specific group. This part of the architecture is discarded after training. The authors of CosPlace create a fixed number of groups from continuous camera poses such that positions and viewpoints within a group are similar but differ strongly between groups. They achieve this by partitioning the position and viewpoint into evenly sized sections. For each training epoch, a different section is considered.

In contrast, our training dataset is already organized into discrete camera poses and the field of view is unlimited due to the panoramic nature of the images. Consequently, we introduce one group per grid node. Because grid sizes vary between training settings, each setting is used with a separate classification network with the number of output units conforming to the number of grid nodes.

We then fine-tune by training for 100 epochs with 200 batches per epoch. Each batch contains 32 samples, which comprise an image and a class label. We follow the original training scheme and modify brightness, contrast, and saturation at random using the ColorJitter function of the PyTorch 2.5.1 library but disable changes of the hue. For each setting, the maximum change is limited to 0.2. Other training and architectural parameters are retained from the original implementation. After each epoch, we assess the recall@10 on the whole validation partition. The model weights of the epoch with the best quality on the validation dataset (with respect to 20 cm and upright images) are retained for testing.

### Hybrid models for spatial verification

In the following, we outline the proposed hybrid architectures for spatial verification and combine a CNN for image preprocessing with either the Visual Compass or MinWarping.

**Fig. 8:**
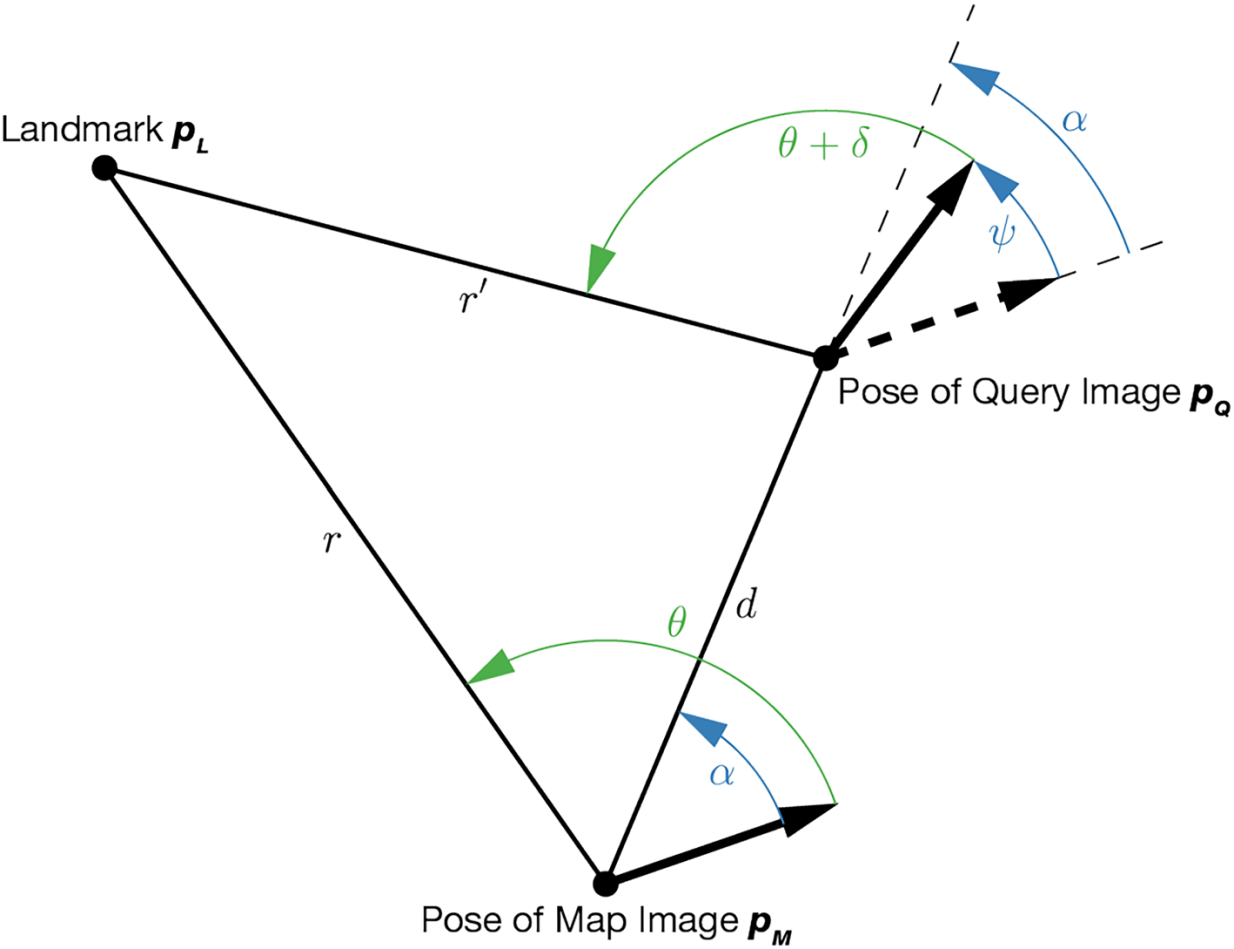
Triangular relation between the position of a landmark $\varvec{p_L}$ and the poses of the query and map images $\varvec{p_Q}, \varvec{p_M}$. The movement parameters estimated by MinWarping ($\alpha , \psi$) are marked blue. The bearing angles to $\varvec{p_L}$ from the forward direction of the robot at $\varvec{p_Q}$ and $\varvec{p_D}$ are marked green. Image based on the work of Möller et al.^7^.

Given a pair of query and map images $(\varvec{Q}, \varvec{M})$, the Visual Compass estimates the 1D rotation $\psi$ between poses $\varvec{p_Q}, \varvec{p_M}$, while MinWarping estimates both the rotation $\psi$ and the bearing angle of the translation between poses, $\alpha$. This is realized by means of the triangular relation between the pose of the landmark $\varvec{p_L}$,the pose of the query $\varvec{p_Q}$, and the pose of the map image $\varvec{p_M}$, under the assumption that every column of a panoramic image is a separate landmark $\varvec{L}$. A visualization is shown in Fig. 8.

We describe map and query images as three-dimensional arrays $\varvec{Q, M} \in [0,1]^{h\times w\times c} \subset \mathbb {R}^{h\times w\times c}$, with $h, w,$ and $c$ as the height (number of rows), width (number of columns), and color channels, respectively. The $i$-th image column is a slice along the second dimension of the image array and is denoted as $\varvec{M}_{1:h,i,1:c}$; a two-dimensional array of size $h \times c$. Both the Visual Compass and MinWarping with coarse-to-fine search are based on the implementation of MinWarping for Tensorflow 2.12 introduced in our previous work^3^.

#### Visual compass

The Visual Compass^6,46^ estimates $\psi$ by computing the pixel-wise distances between the unshifted map image and a set of query images, each one shifted by $i_\psi \in [0,w-1] \subset \mathbb {N}_0$. The rotation with the best fit, $\psi ^*$, belongs to $i_{\psi ^*}$, the index with the least distance.

In this work, we use the modified version of the Visual Compass introduced by Moeller et al.^7^. Here, we compute all column distances simultaneously, creating the distance matrix $\varvec{P}'$ by applying a distance function $d(\varvec{A}, \varvec{B})$ to all combinations of image columns:

1 $$\begin{aligned} \varvec{P}'_{i(\delta ),i(\theta )} = d({\varvec{Q}}_{1: h,i(\theta +\delta ),1:c},{\varvec{M}}_{1:h,i(\theta ),1:c}) \end{aligned}$$

with $\theta , \delta \in [0,2\pi ]$ and $i: [0,2\pi ] \rightarrow [1, ..., w]$ as a function that discretizes angles into column indices. $\theta$ and $\theta +\delta$ are landmark angles with respect to the origin of the image coordinate system. As suggested by Horst and Möller^31^, we use the distance function NSAD:

2 $$\begin{aligned} d(\varvec{A}, \varvec{B}) = \sum _c \frac{\sum _r |\varvec{A}_{r,c}-\varvec{B}_{r,c}|}{\sum _r (|\varvec{A}_{r,c}|+|\varvec{B}_{r,c}|)} \end{aligned}$$

with $r, c$ as indices into the image row and channel. We add $10^{-7}$ to the denominator to prevent undefined values.

The distances $r',r$ shown in Fig. 8 may differ for every landmark. We model this as described by Moeller et al.^7^ by scaling the input images along the vertical axis with the factor $\sigma =\frac{r'}{r}$, using linear interpolation. To this end, we use a fixed number of inversion-symmetric scaling factors $\sigma \in \{0.5, 0.63, 0.79, 1.0, 1.26, 1.59, 2.0\}$ (rounded to two decimal places). For $\sigma < 1$, we upscale $\varvec{M}$ by $\frac{1}{\sigma }$, for $\sigma> 1$, we upscale $\varvec{Q}$ by $\sigma$, and for $\sigma = 1$, we compute $\varvec{P}'$ from unscaled images. This creates a *scale plane stack* $\varvec{P} \in \mathbb {R}^{n_\sigma \times w \times w}$ with $n_\sigma$ as the number of scales. The index of the rotation $i_\psi$ with the least image distance $d_\text {min}$ is then computed as follows:

3 $$\begin{aligned} \varvec{d}&= \sum _k\min _i\varvec{P}_{i,1:w,k}\end{aligned}$$

4 $$\begin{aligned} i_\psi&= \text {argmin}_j\varvec{d}\end{aligned}$$

5 $$\begin{aligned} d_\text {min}&= \text {min}_j \varvec{d} \end{aligned}$$

**Fig. 9:**
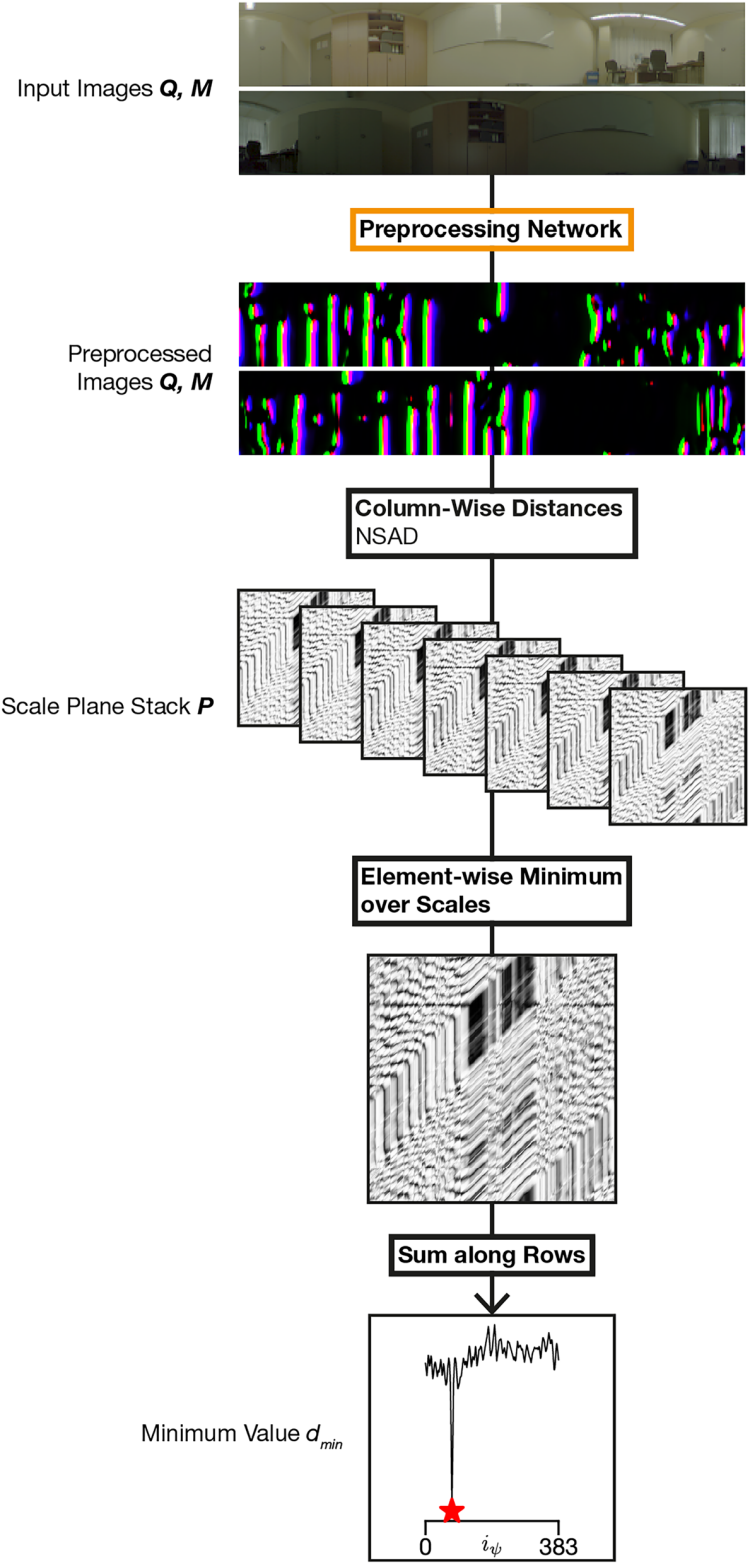
Computation steps of $d_\text {min}$ using the Visual Compass and the proposed network for image preprocessing (orange). The result of the sum along the rows is a vector of distances, represented as a line plot. The minimum is marked with a star.

The final similarity is $-d_\text {min}$. A visualization of the algorithm including the placement of the preprocessing network is shown in Fig. 9.

#### MinWarping

MinWarping extends the Visual Compass to estimate both $\psi$ and $\alpha$ with a geometric model that predicts the landmark movement for a pair of $(\psi , \alpha )$ and an exhaustive search over the discrete steps $i_\alpha \in [0, n_\alpha -1] \subset \mathbb {N}$, $i_\psi \in [0, n_\psi -1] \subset \mathbb {N}$. For a detailed description of the MinWarping algorithm and the geometric model, we refer to the work of Möller et al.^7^.

To achieve comparable discretization to the Visual Compass, we set $n_\alpha , n_\psi$ to the full image width. Saving computation time, we search twice in a coarse-to-fine approach adapted from Möller^47^. The coarse search is run with $n_\alpha = n_\psi = 96$, yielding the indices $i_\alpha ^*$ or $i_\psi ^*$ with the minimum distance. In the fine search, we evaluate the geometric model for $1024$ additional pairs $(i_\alpha , i_\psi )$ drawn from two independent normal distributions each centered at either $i_\alpha ^*$ or $i_\psi ^*$ and a standard deviation of $0.03\cdot w$. We discretize the sampled locations by rounding to integers. Because the indices $i_\alpha , i_\psi$ are cyclic for both $\alpha$ and $\psi$, we take the modulus to achieve wraparound. The negative least distance of the evaluated $i_\alpha , i_\psi$ in the second search $d_\text {min}$ is used as image similarity: $-d_\text {min}$.

**Fig. 10:**
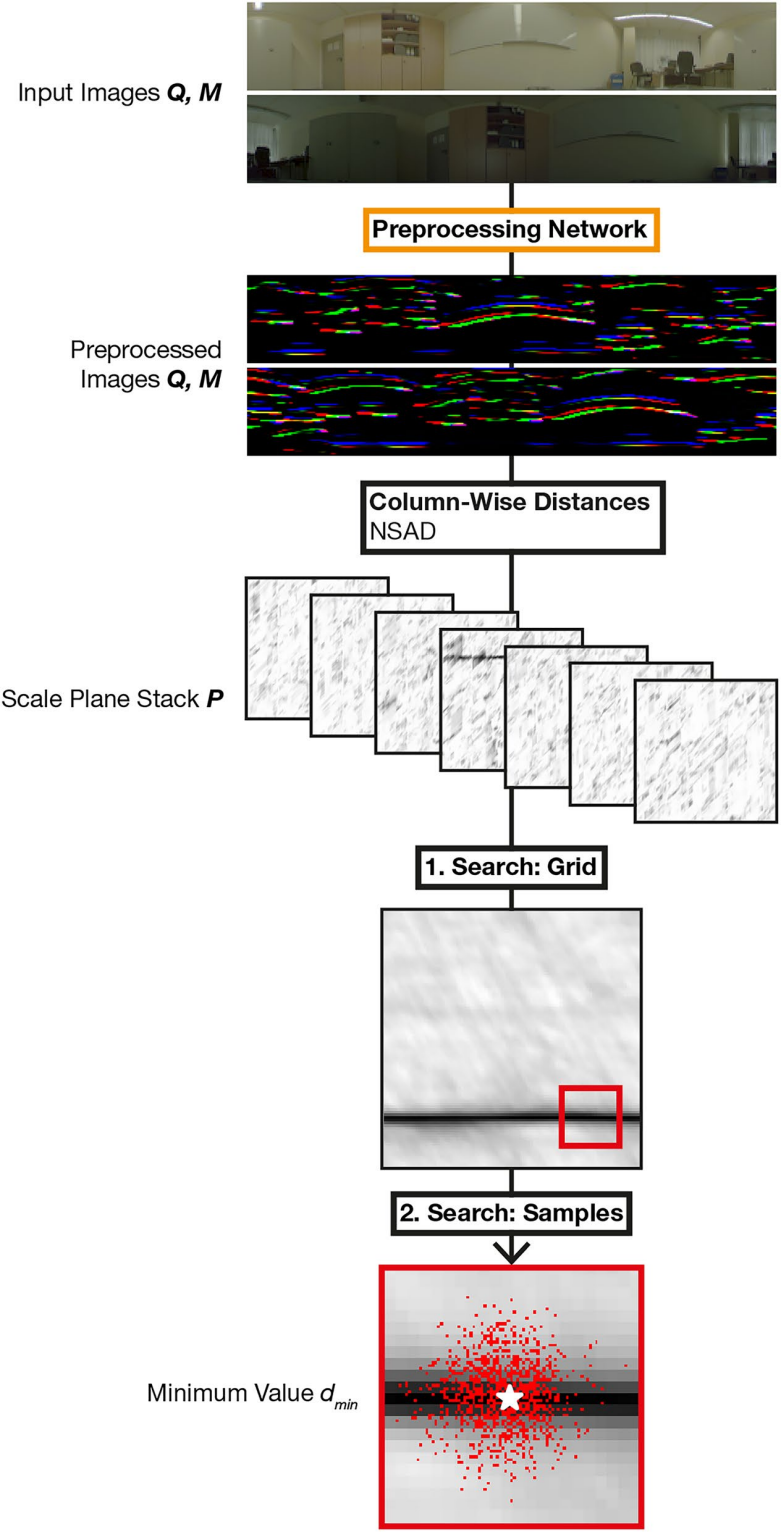
Computation of $d_\text {min}$ using the MinWarping algorithm and the proposed network for image preprocessing. For the second phase of the coarse-to-fine search, a section of the original distance image of the first phase is overlayed with the sample positions (red pixels). The location of the minimum is marked with a star. In this example, we are computing the distance between the query image to a positive example in the map (i.e., an image taken at the same position) and a correct movement direction $\alpha$ cannot be estimated. For an example application of MinWarping for movement estimation, see Fig. 6 in our previous work^3^.

An overview of computation steps for spatial verification with MinWarping, including network-based preprocessing, is shown in Fig. 10.

#### Illumination tolerance: edge filtering as a baseline

As a baseline for illumination tolerance, we apply vertical edge filtering as proposed by Möller et al.^48^ to input images for the Visual Compass and MinWarping. This is realized by convolving an input image $\varvec{I}$ with a $2\times 1$ filter kernel^31,48^

6 $$\begin{aligned} \frac{\partial f}{\partial y} = \begin{bmatrix} -1 \\ +1 \end{bmatrix} * \varvec{I}, \end{aligned}$$

which reduces the image height and the vertical position of the horizon by 1 pixel (measured from the top of the image). Experimental results that refer to the Visual Compass and MinWarping as a baseline include edge filtering, while network preprocessing is supplied with raw RGB color images.

#### Illumination tolerance using hybrid models

**Fig. 11:**
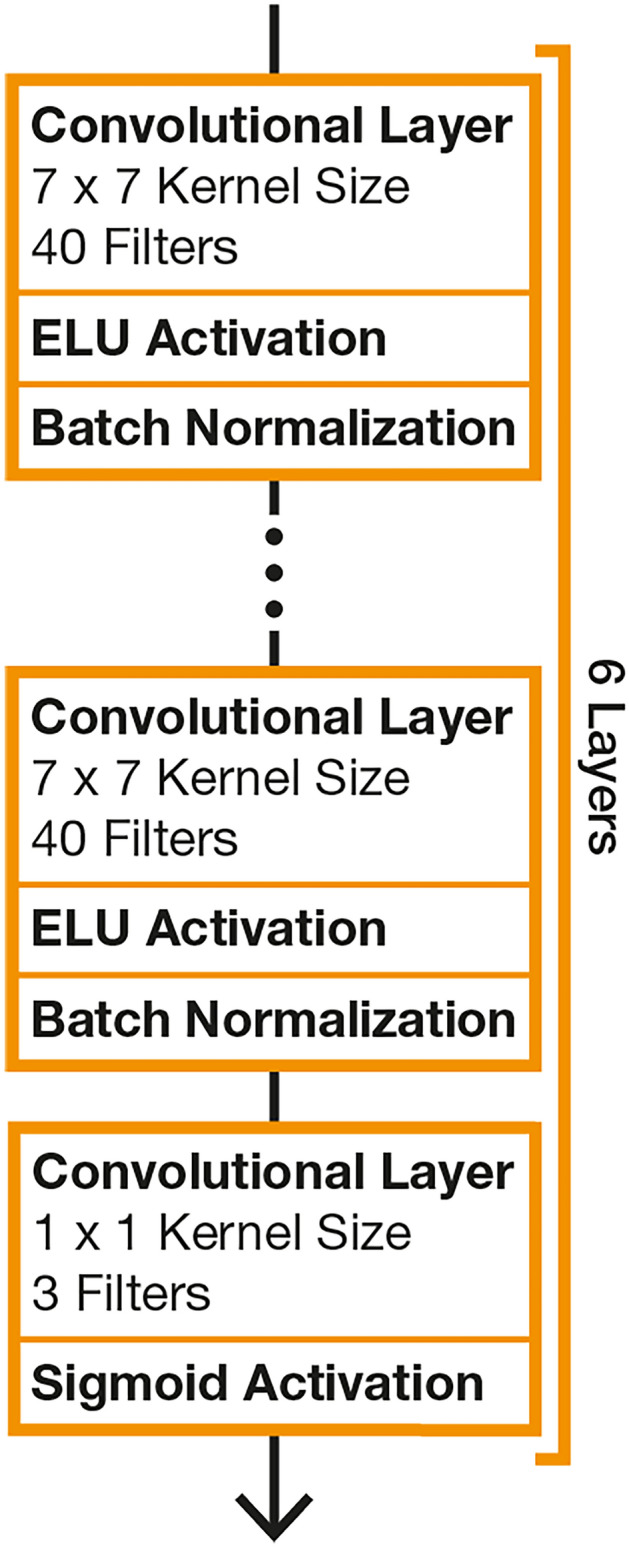
Overview of the network architecture used for image preprocessing. The width of the kernels in the first 6 layers depends on the use of the CNN with a Visual Compass ($K=3$) or MinWarping ($K=5$).

As the preprocessing network for both hybrid models, we use 6 CNN layers with 40 filters of shape $7 \times 7$, ELU activation, and stride 1. An additional final layer with 3 filters of shape $1 \times 1$ and sigmoid activation reduces the number of output channels to 3 and restricts the values to the floatingpoint range [0,1]. A visualization is shown in Fig. 11. The CNN layers use circular padding along horizontal image axis and a reflection of values along the top and bottom row for padding along the vertical axis. The initial weights of the convolutional layers are set using the uniform Xavier initialization^49^ and biases are initialized with zeros. We use batch normalization^50^ after each of the first 6 layers with the default parameters given by Tensorflow 2.12^51^ (momentum = 0.99, $\epsilon = 0.001$, and both learned factors $\beta , \gamma$ enabled). For MinWarping, fine search is enabled during training.

The hybrid MinWarping model for RPE is unchanged from the original work^3^.

We train both hybrid architectures for 30 epochs, each epoch containing 200 batches. A batch is comprised of $m=4$ triplets $(\varvec{Q}_i,\varvec{P}_i,\{\varvec{N}_{i,1},\dots ,\varvec{N}_{i,n}\})$, which contain a query image $\varvec{Q}_i$, a positive example $\varvec{P}_i$, and a set of $n=2$ negative examples $\varvec{N}_{i,j}$. The index $i$ denotes the $i$-th example in the batch. Positive examples are sampled from the same grid node as $\varvec{Q}_i$ but with different illumination. Negative examples are chosen from any variant and from a different grid node to $\varvec{Q}_i$. To provide sufficiently difficult image pairs for training, the distance of the node of $\varvec{N}_{i,j}$ to the correct position is chosen to be at least 2. Within these constraints, all images of a tuple are selected at random.

As the training signal, we use the triplet loss proposed by multiple authors^15,52,53^:

7 $$\begin{aligned} L = \sum _{i=0}^m \sum _{j=0}^n \text {max}(0,d_\text {min}(\varvec{Q}_i,\varvec{P}_i) + \delta -d_\text {min}(\varvec{Q}_i, \varvec{N}_j)). \end{aligned}$$

For each triplet in a batch of size $m$, we simultaneously minimize the distance $d_{\min }$ between $\varvec{Q}_i$ and $\varvec{P}_i$ and maximize the distance between $\varvec{Q}_i$ and $\varvec{N}_i$ until it satisfies a margin $\delta$. To ensure that $d_{\min }$ and $\delta$ lie in the same value range, we divide the distance image by its maximum possible value of three times the image width, yielding $d_{\min } \in [0,1]$. We investigate the influence of $\delta$ on the VPR quality in "Influence of the triplet loss margin on the VPR quality".

After each epoch, we assess the refinement capabilities of the trained model using the validation partition with upright images and 20 cm spacing. To this end, we draw 100 positions from any applicable variant. We then use the trained model to rerank the top $K=10$ images for each drawn position as retrieved by CosPlace. Using the refined ordering of images, we calculate the AUC PR as an estimate of the true AUC PR on the validation partition. This estimate saturates as the training advances. For each hybrid architecture, we select the weights at the last epoch, 30.

### Spatial verification with sparse local features

We implement spatial verification with sparse local features as a pipeline of feature extraction, matching using a neural network, and fitting a geometric model for relative pose estimation (RPE). As a feature extractor, we use SuperPoint^54^ with a maximum of 512 of features per image. We use the implementation and network weights provided by Lindenberger et al.^55^, https://github.com/cvg/LightGlue/tree/main. To establish correspondences between features, we choose SphereGlue^56^ as a neural network architecture specifically designed to match features across a pair of panoramic images, with an implementation and default parameters as provided by the authors (https://github.com/vishalsharbidar/SphereGlue). For SphereGlue and RPE, we convert the feature locations from 2D image coordinates to unit cartesian vectors. Because we remap the fisheye camera images to a section of an ideal sphere using camera intrinsics during dataset preprocessing as described in the section “Datasets”, the angles $\theta , \phi$ for spherical coordinates are computed as

8 $$\begin{aligned} \theta&= \frac{w-x}{w}\cdot 2\pi \end{aligned}$$

9 $$\begin{aligned} \phi&= c + \frac{y}{h}\cdot (o-c) \end{aligned}$$

with $w, h$ as the image width and height, $x, y$ as the horizontal and vertical coordinates of a feature in pixel coordinates, $c$ as the cutoff from the top of the image (corresponding to a fixed angle from the zenith of the image sphere to the top of remaining imaged area after cutoff), and $o$ as the original opening angle from zenith to the bottom of the imaged area (92.5$^{\circ }$).

To create a similarity score for a pair of images, we extract SuperPoint features, map the feature locations to spherical coordinates, and find correspondences using SphereGlue. Then, we estimate the relative camera pose as the rotation $\varvec{R}_{2,1}$ and translation $\varvec{t}_{2,1}$ from camera frame $\varvec{F}_2$, $\varvec{F}_1$ with the method described by Nistér^57^ and outlier rejection using RANSAC. To this end, we use the OpenGV library^58^ with a maximum reprojection error of 0.0003 and at most 1000 RANSAC iterations. RANSAC selects a subset of inliers from the correspondences, i.e. a set of pairs of spherical coordinates

10 $$\begin{aligned} S = \{(\varvec{p}_{1,1},\varvec{p}_{2,1}), \dots (\varvec{p}_{1,i},\varvec{p}_{2,i})\}. \end{aligned}$$

Commonly, the number of inliers is used as a similarity score^4,8–11^. Due to the small physical distance between capture positions and the open environments in our datasets, the inlier count is consistently high with only small variations and therefore too similar (see “Results”). As a mitigation, we use the relative pose estimate to align corresponding points on the sphere and calculate the similarity metric based on the log mean squared error:

11 $$\begin{aligned} \text {sim}(S) = {\left\{ \begin{array}{ll} -\log \Big (\epsilon + \frac{1}{|S|}\sum _{(\varvec{p}_1, \varvec{p}_2) \in S} (\varvec{p}_1 - \varvec{R}_{2,1}\varvec{p}_2)^2 \Big ) & \text {for } |S|> 0 \\ - \infty & \text {for } |S| = 0 \end{array}\right. } \end{aligned}$$

with $\epsilon = 10^{-7}$ to prevent undefined values in the case of perfectly aligned point pairs $(\varvec{p}_1, \varvec{p}_2)$.

**Fig. 12:**
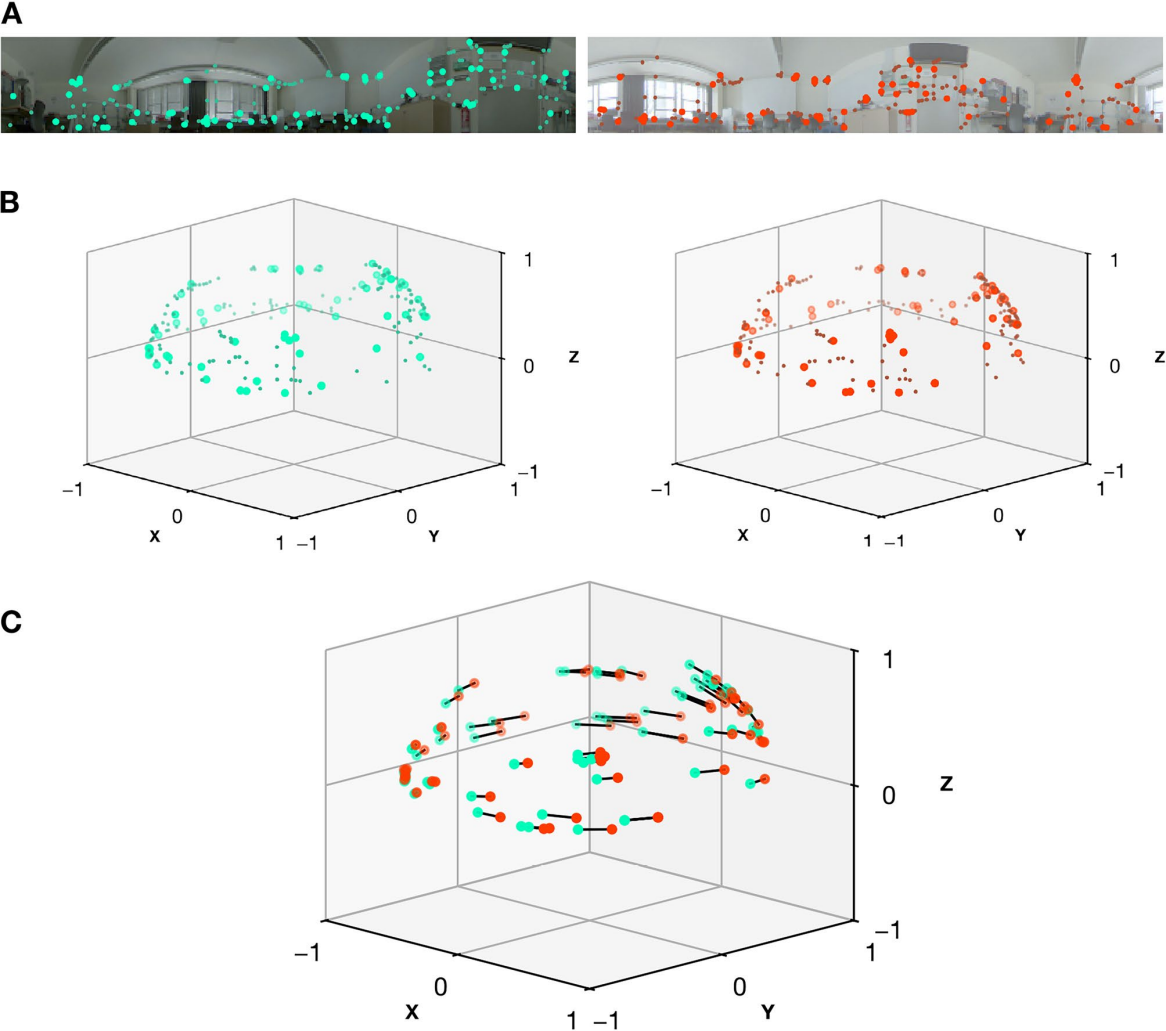
Overview of alignment of the sets of detected features for two images of the Robotics Lab setting (Fish dataset). The left image was captured at the grid origin, while the right image was captured about 60 cm in direction of the y-axis. *A:* We detect and describe features in both images using SuperPoint^54^. We then randomly select 70 of these features to improve visibility during the matching step. These features are displayed using larger and brighter markers. (**B**) To allow matching with SphereGlue^56^ and to support the RANSAC^29^ implementation in OpenGV^58^, feature coordinates are mapped to the unit sphere. Due to the camera setup and the image processing, only a section of the upper hemisphere is used. (**C**) After finding correspondences with SphereGlue, we use Nistér’s five-point algorithm^57^ and RANSAC to estimate the 6D relative pose of the second (right) camera frame. The relative rotation is then used to align the second to the first camera frame. In this example, the pose estimation is close to the correct solution, and the alignment works well. The translation between poses means feature points coincide if they are near the focus of expansion or focus of contraction, but they lie farther apart for points in between. Our proposed metric builds upon the sum of the euclidean distances between the points, indicated by black lines.

After alignment, the shift of local features induced by the camera motion is an indicator of the translation between views, and therefore, the image similarity. An overview of this process is shown in Fig. 12. Note that our approach is conceptually similar to the Visual Compass, but instead of estimating the correct alignment using a pixel-wise error, we are relying on a geometric error.

## Results

In the following, we investigate the place recognition quality of the proposed hybrid architectures and compare with VPR based on sparse local features. As a precondition to spatial verification, we first report on the achieved recall with CosPlace.

### Recall with CosPlace

**Fig. 13:**
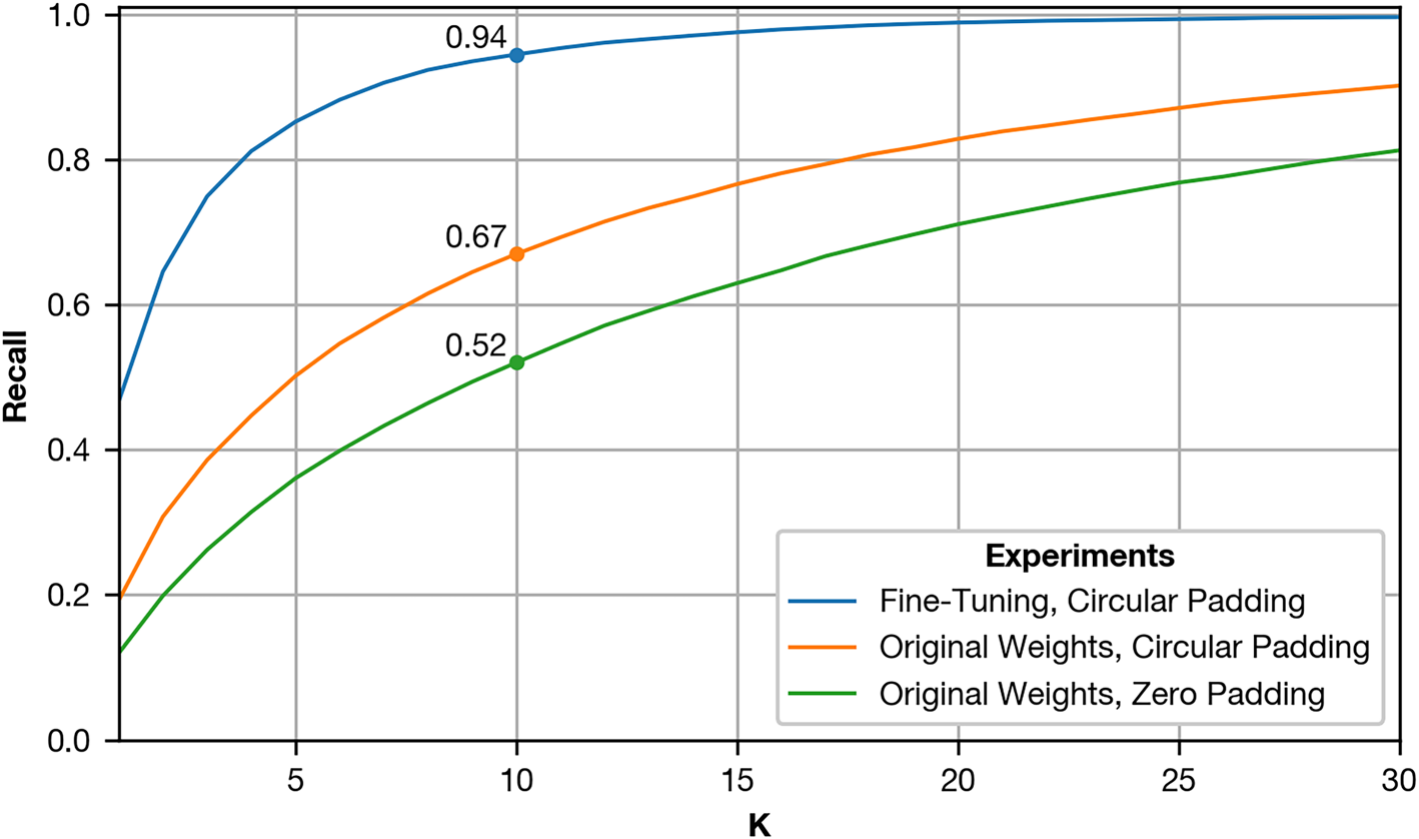
Effects of circular padding and fine-tuning on the recall when retrieving images with CosPlace. We show the recall for K up to 30 and display the numerical values for $K=10$, rounded to two decimal places. The recall is assessed on the validation partition for upright images and with a 20 cm grid spacing.

As shown in Fig. 13, both fine-tuning and circular padding improve the recall on the validation partition over CosPlace with default weights and are enabled for all future experiments. For details on the implementation of these modifications, see "Image retrieval with CosPlace". Following these results, we retrieve $K= 10$ candidates for all following experiments as a tradeoff between the retrieval quality and the computational effort added by spatial verification.

**Fig. 14:**
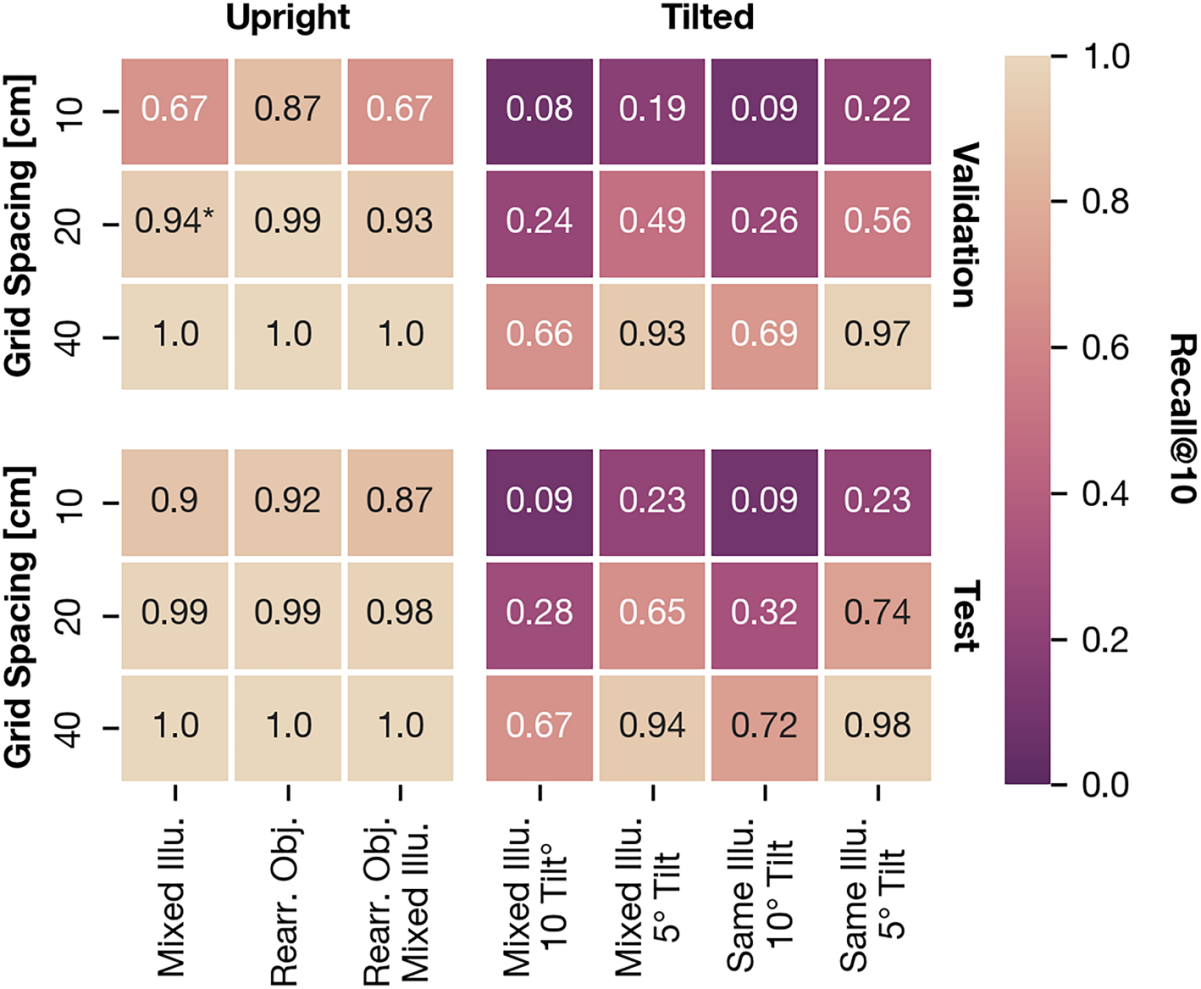
Recall@10 for CosPlace on the validation (upper row) and test partition (lower row). All values are rounded to two decimal places. Results are split into variant pairs with upright camera orientation (right column) and pairs with tilt (left column). Within each heatmap, each row corresponds to an instance of grid spacing. All cells show results for Robotics Lab II, except the one marked with an asterisk (20 cm spacing, upright images, validation partition). For this cell, the additional data of Robotics Lab is available, and we show the average between both settings. This is consistent with the results shown in Fig. 13.

The recall@10 afforded by CosPlace is also the upper bound of the PR AUC for spatial verification. In Fig. 14, we quantify this limit for the validation and test partitions. Following this, CosPlace is robust against illumination changes, rearrangement of objects, and 5$^{\circ }$ tilt with and without illumination changes. The recall@10 decreases for finer grid spacing, especially 10 cm, and 10$^{\circ }$ tilt.

### Influence of the triplet loss margin on the VPR quality

To select a suitable margin $\delta$ for the triplet loss, we perform a linear parameter search by training the hybrid Visual Compass with $\delta \in \{0.2, 0.4, 0.6, 0.8, 1.0\}$. Note that for the excluded case $\delta = 0.0$, optimization does not continue once positives are as close to the query as negatives ($d_\text {min}(\varvec{Q}_i,\varvec{P}_i) \approx d_\text {min}(\varvec{Q}_i, \varvec{N}_j)$), causing poor separation.

**Fig. 15:**
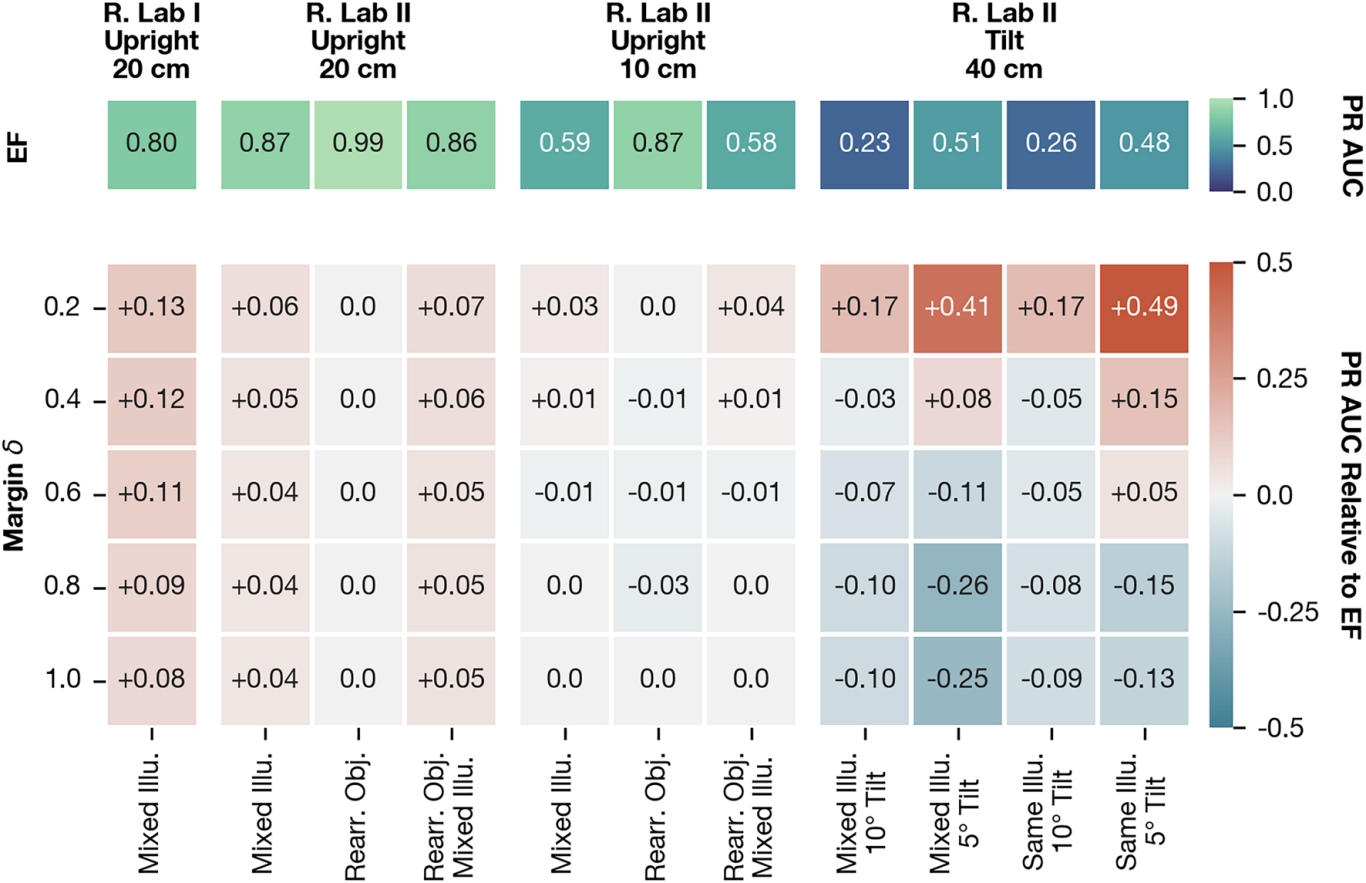
Influence of the margin $\delta$ on the PR AUC assessed on the validation partition for the hybrid Visual Compass. The first row shows PR AUC values for the Visual Compass with edge filtering (EF). Subsequent rows represent the difference between the PR AUC of the hybrid model and Visual Compass with EF, with positive values corresponding to an improvement of VPR quality over the baseline. Each column refers to an appearance subset (see “Experimental setup”). Columns are grouped by setting, the use of camera tilt, and the grid spacing. For Robotics Lab II with upright camera orientation and a grid spacing of 40 cm, the Visual Compass with NP reaches a perfect score of 1.0 regardless of the set margin (data omitted).

Figure 15 shows the influence of $\delta$ on the VPR quality on the validation partition in comparison to the baseline, the Visual Compass with edge filtering. We observe the best overall quality for $\delta =0.2$. For this choice, the PR AUC is moderately improved for upright images and substantially improved for tilted images. After confirming that this margin is also suitable for MinWarping, we select it for all future experiments for both models.

**Fig. 16:**
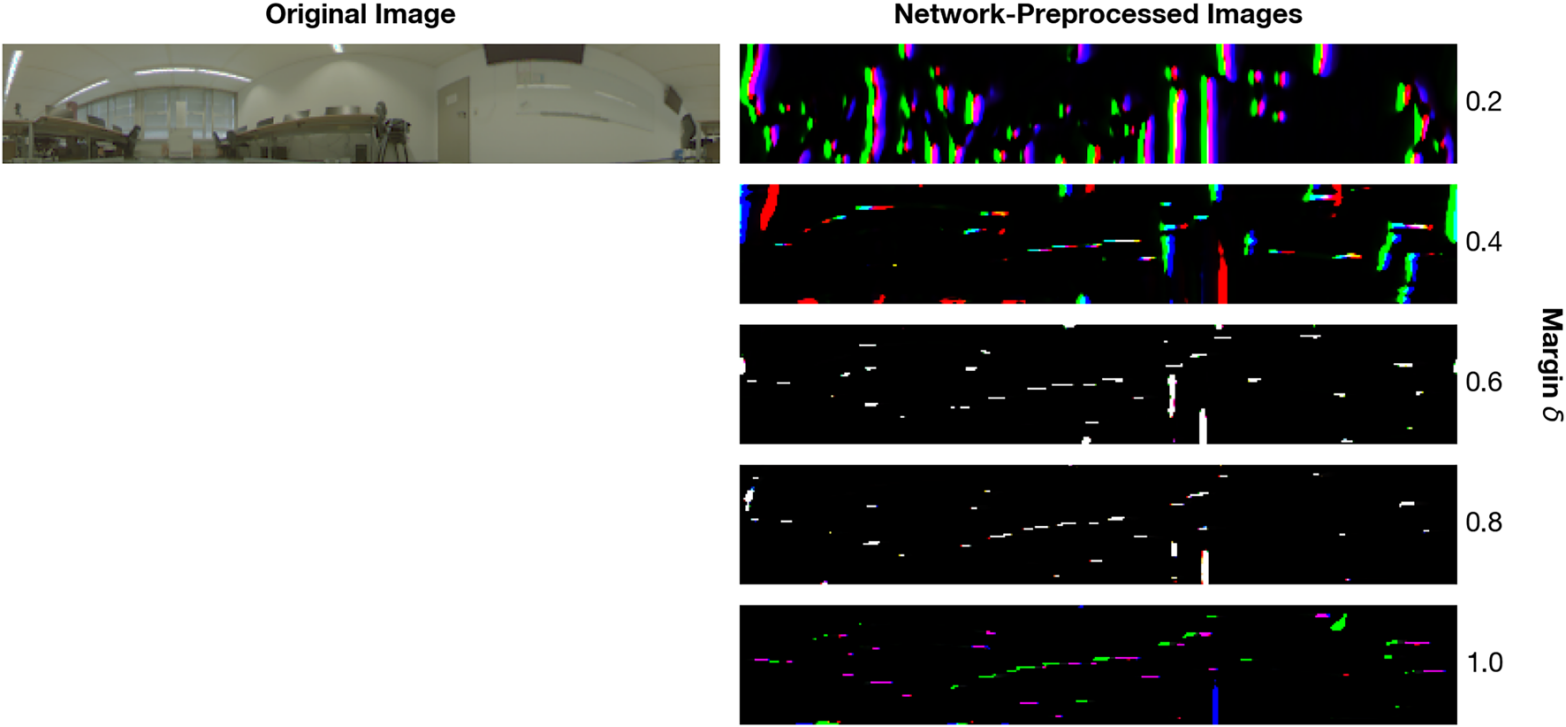
Overview of network-preprocessed images for the Visual Compass with different margins set for the triplet loss (right column). The original image (left column) stems from the middle of the capture area of the setting Gantry Lab and the variant Lights. The three output channels of the preprocessing network are interpreted as red, green, and blue colors, respectively.

To investigate the variation in VPR quality for tilted images, we look at examples of processing results for each choice of $\delta$ (see Fig. 16). Common to all solutions are sparse responses with uniform colors; a reaction of the network to salient features in the input image. Notably, responses for training with small margins $\delta$ have an elongated shape in the vertical direction. As the margin increases, responses become shorter up to a minimal height of a single pixel. We further discuss this observation in the section “Discussion”.

### VPR quality with hybrid models and sparse local features

**Fig. 17:**
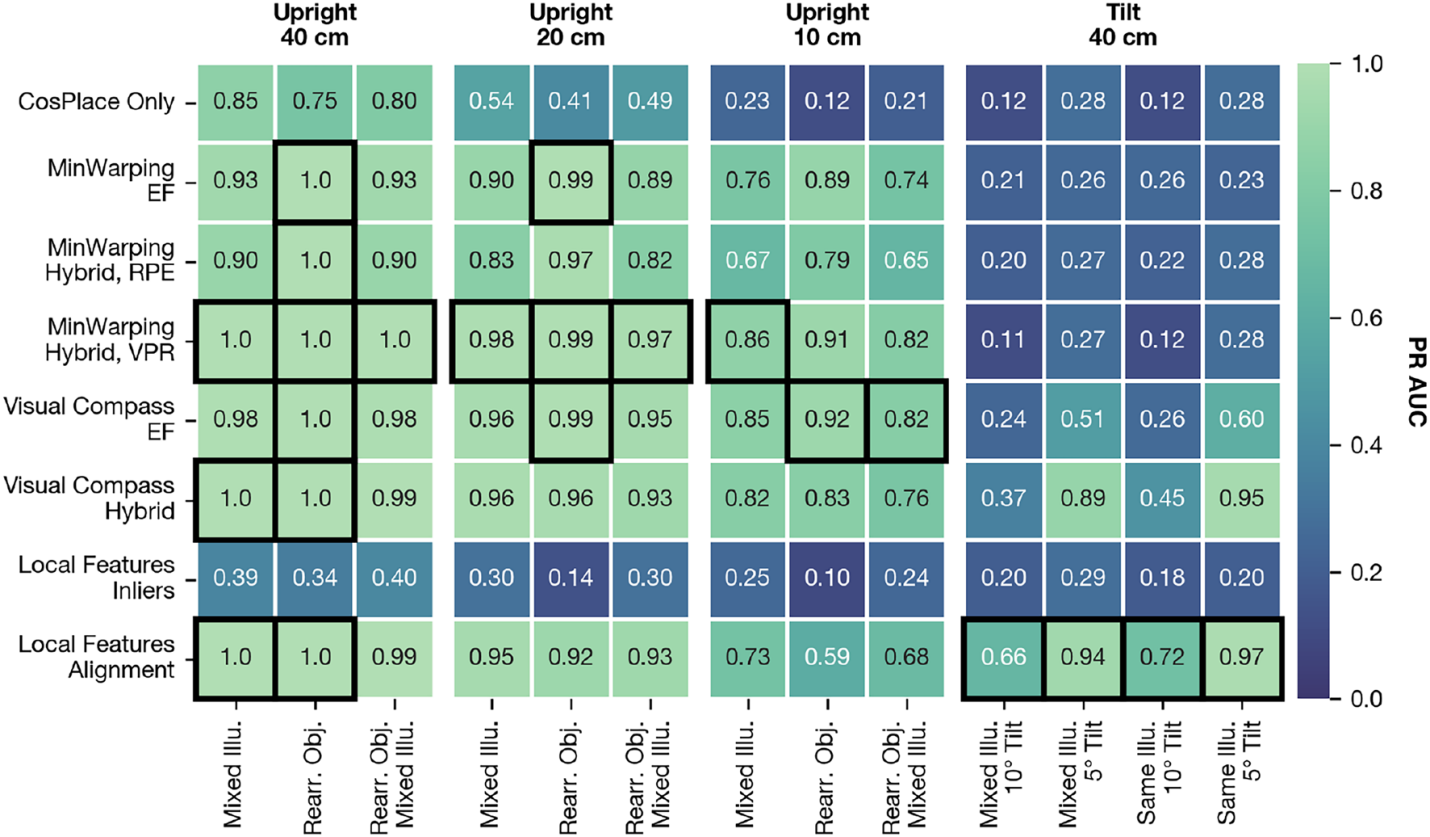
PR AUC assessed on the test partition for hybrid models, edge filtering baselines, and sparse local features (abbreviated to “Local Features”). Each column group shows results for an instance of grid spacing for upright or tilted images. The best results for each column are marked with a black box. All values are rounded to two decimal places.

In Fig. 17, we compare the PR AUC for the Visual Compass, MinWarping, and sparse local features on the test partition. As an additional baseline, we show results for CosPlace without spatial verification. We distinguish between preprocessing with edge filtering (abbreviated as *EF*) and hybrid models. For the latter, we consider variants specifically trained for place recognition (VPR; with the margin $\delta = 0.2$) and relative pose estimation (RPE). Sparse local features are tested with inliers and alignment-based scoring.

In situations with an upright camera orientation, most methods substantially improve the precision over just using image retrieval with CosPlace. Here, illumination changes are more challenging than rearranged objects, and finer grid resolutions are more challenging than coarser ones. The VPR quality for camera tilt is low for most of the tested methods.

The optimal VPR quality is achieved by both purposefully trained hybrid models, the Visual Compass with EF, and sparse local features with alignment. For MinWarping and the Visual Compass, the quality for upright images is similar, but the Visual Compass offers better tilt tolerance. Here, the hybrid Visual Compass substantially improves upon EF; however, the PR AUC for this method is slightly worse for upright images with 10 cm spacing if objects are rearranged.

Note that due to data availability, network-based models are only trained on upright images with a spacing of at least 20 cm and are therefore not optimized for tilt tolerance or VPR quality with 10 cm spacing.

Comparing MinWarping hybrid models, we observe that the purposefully trained network improves upon edge filtering for upright images, leading to optimal VPR quality in these circumstances. When hybrid MinWarping is trained for RPE, the VPR quality is lower than the baseline (MinWarping with EF).

Sparse local features with inlier-based scoring offers poor VPR quality for the tested environments and is the only method that reduces the precision over just using CosPlace. We discuss possible reasons in the following section “Discussion”. The proposed alignment-based scoring is competitive with our hybrid models for upright images with the exception of 10 cm grid spacing. For image pairs with 10$^{\circ }$ tilt, it is the best suited method, and it also slightly improves the VPR quality for 5$^{\circ }$ tilts over the hybrid Visual Compass.

## Discussion

Our observations indicate that the image processing requirements for the applications RPE and VPR differ substantially.

**Fig. 18:**
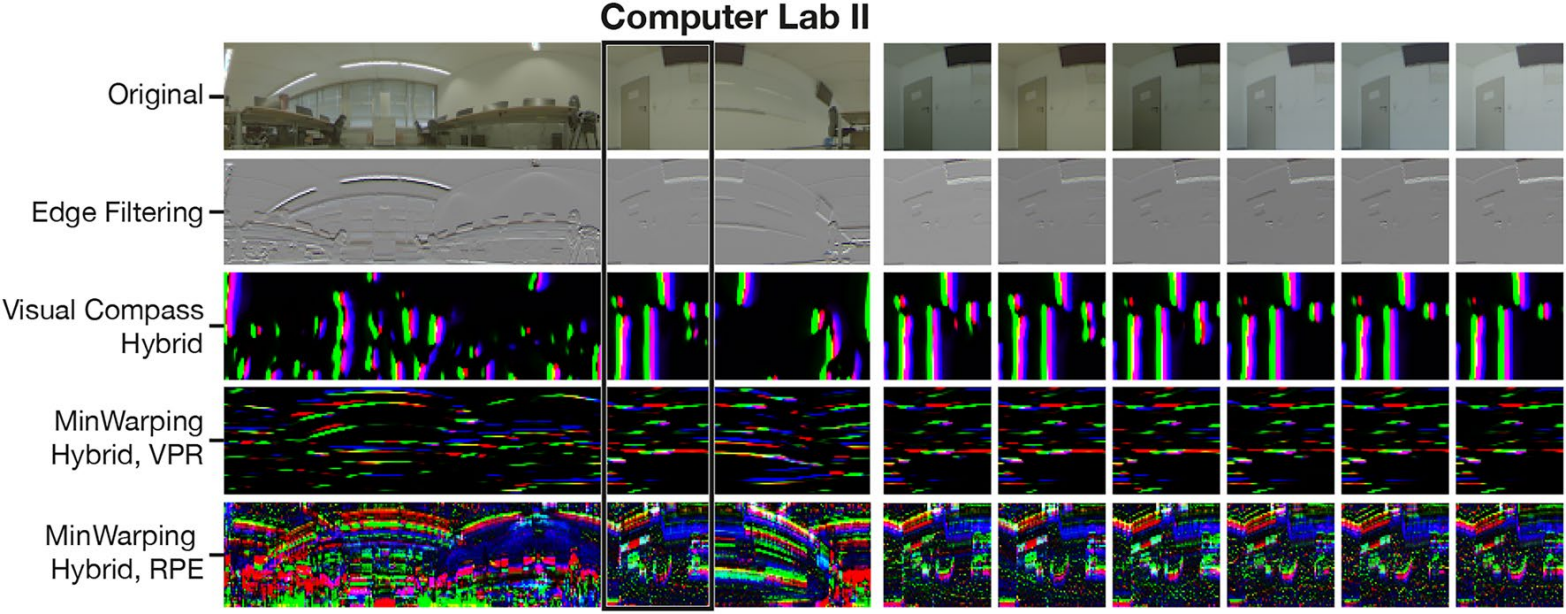
Comparison of image preprocessing for the setting Computer Lab II. The leftmost column shows the full-sized image taken at the center of capture area ($x=17, y=31$ considering 5 cm grid spacing) for the variant *Lights*. For the sections marked with the black box, we show preprocessing results for different variants. In particular, the six columns on the right show the variants *No Lights*, *Lights Close*, *Lights Far*, *Rainy*, *Sunny Morning*, and *No Shutters Cloudy* from left to right. Each row corresponds to an image preprocessing method. The topmost row shows the unprocessed image used as input for any filtering method. The second row represents the result of the edge filter, with values scaled to the range [0,1]. The last three rows show the output of CNN-based preprocessing for the Visual Compass, MinWarping for VPR, and MinWarping for RPE. To this end, the three output channels are interpreted as RGB colors.

For each task, the specific training can improve the quality over using edge filtering, but transferring the preprocessing from RPE to VPR leads to worse results. To further investigate, we compare preprocessing results for edge filtering and hybrid models in Fig. 18. Network preprocessing for RPE creates dense, finely structured responses. In our previous work^3^, we identified two causes: first, salient features are marked more prominently in comparison to edge filtering, and second, the network amplifies noise in regions that lack salient features, which reduces their influence in the final pose estimate. Both effects allow a more robust matching of columns between images, an important prerequisite for estimating the pose accurately.

In contrast, we observe sparsely distributed filter responses with coarser structures when training the hybrid models for VPR. Even though the application is the same, the preprocessing solutions are markedly different between the Visual Compass and MinWarping. For the former, the preprocessing is characterized by the margin selected for the triplet loss (see also "Influence of the triplet loss margin on the VPR quality"). If $\delta$ is small, the hybrid model relies on large, vertical structures, but if $\delta$ is large, markers become as short as 1 px in height. This can be explained by viewing the preprocessing as an embedding of images into a feature space. The embedding is optimized such that distances within the feature space for samples belonging to same position are small regardless of illumination. At the same time, distances between embeddings for different positions must satisfy the margin. When larger margins are enforced, successful training increases the space between examples in the embedding if they represent different places. By design of the network, entries of the embedding must be in the range [0,1] (see "Illumination tolerance using hybrid models"). Therefore, the largest distance between two embeddings is achieved if the entries of the first image are close to the smallest possible value, 0.0, in the place where the other has the largest possible value, 1.0. This leads to reduced feature size and increased sparsity.

In contrast, hybrid MinWarping operates on slim, horizontal responses. Regardless of the choice of $\delta$, we observed patterns similar to the ones shown in Fig. 18 with regards to the sparsity, the shape, and the distribution of information to color channels. Because the MinWarping algorithm is a superset of the Visual Compass, this behavior must be caused by the additional search for the movement parameter $\alpha$.

For successful VPR, the distance between compared images $d_{\min }$ should be consistently lower for image pairs at the same position when compared to pairs representing a change in position. However, MinWarping is designed to not only tolerate but actively use visual cues of the translation between views for robust RPE. Training network preprocessing to reduce this translation tolerance is vital to achieve ideal VPR results.

When capturing panoramic images, we are applying a spherical camera model to map fisheye images to an equirectangular format (see “Dataset collection”). In the spherical model, any physical displacement of the camera causes the image content to move along great circles. These great circles pass through the focus of expansion (FOE), where motion appears to originate, and the focus of contraction (FOC), where motion seems to vanish. When mapping the fisheye image to the equirectangular format, the great circle that coincides with the movement direction is represented as a straight line between FOE and FOC. Above and below this line, image content moves along arcs of increasing curvature. In terms of our hybrid models, even small displacements of the camera cause responses to shift along these arcs. Due to the short height of the filter responses of hybrid MinWarping, vertical movement causes them to misalign within compared images, which leads to large increases of the final image distance. Therefore, the specific shape of the responses reduces the translation tolerance of MinWarping and leads to better VPR results for upright images.

In contrast, the VPR quality of MinWarping is worse than the one of the Visual Compass if both methods are using edge filtering (see "VPR quality with hybrid models and sparse local features"). This shows that the added invariance to translation of MinWarping can impair performance if the preprocessing is not task-specific. However, it appears beneficial for the training of the hybrid model, because hybrid MinWarping is competitive and even improves upon the hybrid Visual Compass for upright images.

**Fig. 19:**
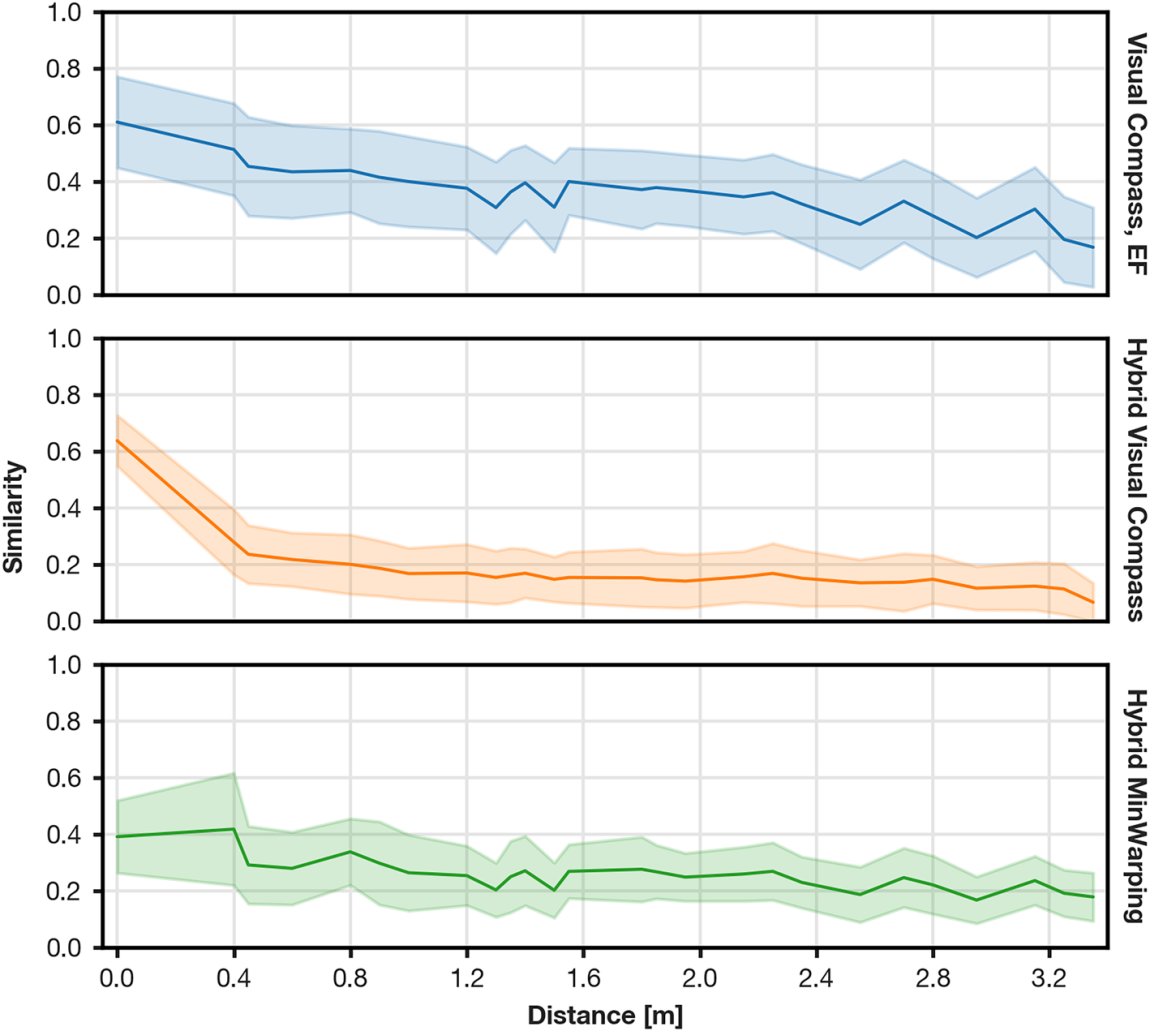
Comparison of the image similarity in relation to the physical distance of capture points on the test partition for 5$^{\circ }$ tilts (mixed and same illumination). Similarities are binned by distance; every bin is 5 cm wide. The mean similarity is shown as a solid line and the standard deviation is represented by the transparent area. Hybrid MinWarping is trained for VPR.

Next, we observed that most tested methods are sensitive to camera tilt. This is by design: both the Visual Compass and MinWarping assume movement in the plane. Despite not being trained with tilted images, the hybrid Visual Compass improves tilt sensitivity substantially. Network preprocessing alone does not guarantee tilt tolerance, as hybrid MinWarping is less robust in this category. To further investigate, we show the relation between physical distance and image similarity for the Visual Compass with EF and both hybrid models Fig. 19. Here, we are especially interested in the reduction of similarity between the same position and the neighboring grid nodes (distance 0.4 m). For the Visual Compass with either EF or network preprocessing, similarities generally decrease. The drop-off is noticeably steeper for the hybrid model, which facilitates classification between images at the same and neighboring positions. For hybrid MinWarping, the similarity of neighboring nodes is on average higher than for the same position, leading to many false positives. We also observed this behavior for MinWarping with EF and hybrid MinWarping for RPE.

The improved tilt robustness for the hybrid Visual Compass is caused by the distinct vertical shapes of the filter response. To classify image pairs correctly, the image similarity after distortion due to tilt must be higher than the similarity after distortion due to movement. Movement introduces a displacement of features along the great circles between FOE and FOC. Tilt displaces filter responses along the elevation direction of the spherical coordinate system and large, vertical responses offer a larger overlap.

**Fig. 20:**
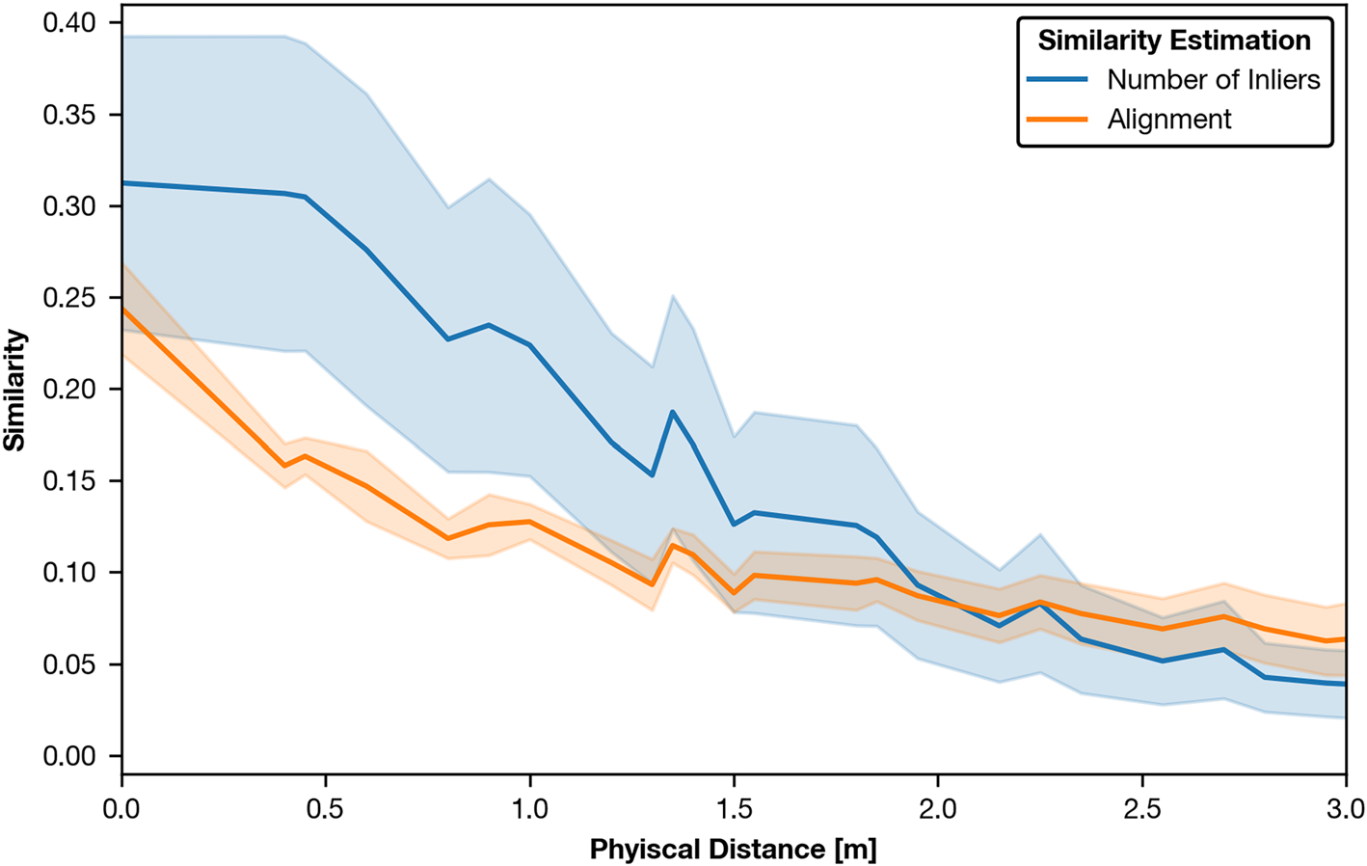
Comparison of the relation between the physical distance and the similarity between compared images as estimated using the number of inliers or the alignment-based scoring. For visualization, similarity values are binned by physical distance into bins of 5 cm width. The solid line represents the mean and the shaded area shows the standard deviation of samples at the given bin. This example uses data from the test partition for upright images and appearance changes (illumination and rearranged objects). Similarity values are normalized to the range [0,1] per-setting and over all variant pairs, including the ones not used for this evaluation.

Lastly, we showed that the inlier count is not well suited for spatial verification with sparse local features in the presented application. In Fig. 20, we compare the distribution of similarities between scoring based on the number of inliers and alignment for 40 cm grid spacing and show that the number of inliers exhibits high variance and only decreases reliably for distances larger than 40 cm, causing the VPR system to select neighboring points instead of the correct place. In contrast, the alignment-based similarity metric has less variance and differs meaningfully between the same and neighboring positions, allowing for more robust place recognition. We expect the suggested alignment score to perform well as long as the estimation of the relative pose is well supported by a high inlier count and local features are well distributed throughout the panoramic image.

Despite the improvement provided by the alignment-based scoring, our proposed hybrid models compare favorably with VPR based on sparse local features for movement in the plane and finer grid spacings of 20 cm and 10 cm. The full 6D pose estimation afforded by the five-point algorithm is especially advantageous for tilted viewpoints, where sparse local features provide the best VPR quality out of the compared methods.

## Conclusion

We presented and compared hybrid machine learning models for visual place recognition on the basis of MinWarping and the Visual Compass. For MinWarping, we reported an improved tolerance for illumination changes and rearranged objects when compared to the baseline, edge filtering, yielding the best results for upright images in our test. In comparison to the Visual Compass, using the additional search for movement parameters during training may assist in discriminating images that are physically close. This is beneficial in applications that demand a low false positive rate of VPR, e.g. during loop closure in SLAM systems.

For the Visual Compass, we observed a drastically improved tilt tolerance in comparison to all other models in the test, despite not being designed or trained for it. This improvement is caused by the distinct shape of the filter responses that still align well after small tilts of the camera (up to 5$^{\circ }$). While the VPR quality for this model for upright images is still competitive, it falls behind MinWarping for VPR and the Visual Compass with edge filtering. Due to the good tilt tolerance, the hybrid Visual Compass is still one of the most versatile approaches in our test. Following these results, we suspect that further diversification of the training dataset may provide additional improvements, e.g. including tilted images. Another possibility for future work may include building a hybrid model using a 3D Visual Compass^59^, which may provide more robust estimates of the image distance after camera tilt. Finally, training and verifying the proposed hybrid models with datasets of a larger variety of indoor environments and outdoor scenes may yield additional insights into the generalizability of the proposed hybrid models.

## Supplementary Information

Supplementary Information.

## Data Availability

The processed image databases, the weights of the hybrid models for VPR, and source code for training are available at https://gitlab.ub.uni-bielefeld.de/loffermann/HybridVPR.

## References

[1] Iscen, A., Tolias, G., Avrithis, Y., Furon, T. & Chum, O. Panorama to panorama matching for location recognition. In *Proceedings of the 2017 ACM on International Conference on Multimedia Retrieval*, 392–396 (2017).

[2] Gao, S., Yang, K., Shi, H., Wang, K. & Bai, J. Review on panoramic imaging and its applications in scene understanding. *IEEE Transactions on Instrumentation and Measurement* **71**, 1–34 (2022).

[3] Offermann, L. Relative pose estimation from panoramic images using a hybrid neural network architecture. *Scientific Reports* **14**, 25246. 10.1038/s41598-024-75124-7 (2024). 39448675 10.1038/s41598-024-75124-7 PMC11502857

[4] Masone, C. & Caputo, B. A survey on deep visual place recognition. *IEEE Access* **9**, 19516–19547. 10.1109/ACCESS.2021.3054937 (2021).

[5] Berton, G., Masone, C. & Caputo, B. Rethinking visual geo-localization for large-scale applications. In *2022 IEEE/CVF Conference on Computer Vision and Pattern Recognition (CVPR)*, 4868–4878. 10.1109/CVPR52688.2022.00483 (IEEE, New Orleans, LA, USA, 2022).

[6] Zeil, J., Hofmann, M. I. & Chahl, J. S. Catchment areas of panoramic snapshots in outdoor scenes. *Journal of the Optical society of America A* **20**, 450–469 (2003). 10.1364/josaa.20.000450 12630831

[7] Möller, R., Krzykawski, M. & Gerstmayr, L. Three 2D-warping schemes for visual robot navigation. *Autonomous Robots* **29**, 253–291 (2010).

[8] Lee, S., Seong, H., Lee, S. & Kim, E. Correlation verification for image retrieval. In *2022 IEEE/CVF Conference on Computer Vision and Pattern Recognition (CVPR)*, 5364–5374. 10.1109/CVPR52688.2022.00530 (IEEE, New Orleans, LA, USA, 2022).

[9] Hausler, S., Garg, S., Xu, M., Milford, M. & Fischer, T. Patch-NetVLAD: Multi-scale fusion of locally-global descriptors for place recognition. In *2021 IEEE/CVF Conference on Computer Vision and Pattern Recognition (CVPR)*, 14136–14147. 10.1109/CVPR46437.2021.01392 (IEEE, Nashville, TN, USA, 2021).

[10] Valgren, C. & Lilienthal, A. Incremental spectral clustering and seasons: Appearance-based localization in outdoor environments. In *2008 IEEE International Conference on Robotics and Automation*, 1856–1861. 10.1109/ROBOT.2008.4543477 (2008).

[11] Ramisa, A., Tapus, A., Aldavert, D., Toledo, R. & Lopez de Mantaras, R. Robust vision-based robot localization using combinations of local feature region detectors. *Autonomous Robots* **27**, 373–385 (2009).

[12] Sivic & Zisserman. Video Google: a text retrieval approach to object matching in videos. *In Proceedings Ninth IEEE International Conference on Computer Vision* **2**, 1470–1477. 10.1109/ICCV.2003.1238663 (2003).

[13] Jégou, H., Douze, M., Schmid, C. & Pérez, P. Aggregating local descriptors into a compact image representation. In *2010 IEEE Computer Society Conference on Computer Vision and Pattern Recognition*, 3304–3311. 10.1109/CVPR.2010.5540039 (2010).

[14] Jaakkola, T. & Haussler, D. Exploiting generative models in discriminative classifiers. In Vol. 11 (eds Kearns, M., Solla, S. & Cohn, D.) *Advances in Neural Information Processing Systems*, (MIT Press, 1998).

[15] Arandjelovic, R., Gronat, P., Torii, A., Pajdla, T. & Sivic, J. NetVLAD: CNN architecture for weakly supervised place recognition. *IEEE Transactions on Pattern Analysis and Machine Intelligence* **40**, 1437–1451. 10.1109/TPAMI.2017.2711011 (2018). 28622667 10.1109/TPAMI.2017.2711011

[16] Keetha, N. et al. Anyloc: Towards universal visual place recognition. *IEEE Robotics and Automation Letters* (2023).

[17] Tzachor, I. et al. EffoVPR: Effective foundation model utilization for visual place recognition, 10.48550/ARXIV.2405.18065 (2024).

[18] Lu, F. et al. Towards seamless adaptation of pre-trained models for visual place recognition. In *The Twelfth International Conference on Learning Representations* (2024).

[19] Oliva, A. & Torralba, A. Building the gist of a scene: The role of global image features in recognition. *Progress in brain research* **155**, 23–36 (2006). 17027377 10.1016/S0079-6123(06)55002-2

[20] Dalal, N. & Triggs, B. Histograms of oriented gradients for human detection. In *2005 IEEE computer society conference on computer vision and pattern recognition (CVPR’05)*, **1**, 886–893 (Ieee, 2005).

[21] Menegatti, E., Maeda, T. & Ishiguro, H. Image-based memory for robot navigation using properties of omnidirectional images. *Robotics and Autonomous Systems* **47**, 251–267. 10.1016/j.robot.2004.03.014 (2004).

[22] Ali-Bey, A., Chaib-Draa, B. & Giguere, P. MixVPR: Feature mixing for visual place recognition. In *Proceedings of the IEEE/CVF winter conference on applications of computer vision*, 2998–3007 (2023).

[23] Berton, G., Trivigno, G., Caputo, B. & Masone, C. EigenPlaces: Training viewpoint robust models for visual place recognition. In *2023 IEEE/CVF International Conference on Computer Vision (ICCV)*, 11046–11056, 10.1109/ICCV51070.2023.01017 (IEEE, Paris, France, 2023).

[24] Shi, Z. et al. Panovpr: Towards unified perspective-to-equirectangular visual place recognition via sliding windows across the panoramic view. In *2023 IEEE 26th International Conference on Intelligent Transportation Systems (ITSC)*, 1333–1340 (IEEE, 2023).

[25] Gard, N., Hilsmann, A. & Eisert, P. Spvloc: Semantic panoramic viewport matching for 6d camera localization in unseen environments. In *European Conference on Computer Vision*, 398–415 (Springer, 2024).

[26] Noh, H., Araujo, A., Sim, J., Weyand, T. & Han, B. Large-scale image retrieval with attentive deep local features. In *2017 IEEE International Conference on Computer Vision (ICCV)*, 3476–3485. 10.1109/ICCV.2017.374 (IEEE, Venice, 2017).

[27] Galvez-López, D. & Tardos, J. D. Bags of Binary Words for Fast Place Recognition in Image Sequences. *IEEE Transactions on Robotics* **28**, 1188–1197. 10.1109/TRO.2012.2197158 (2012).

[28] Hartley, A. & Zisserman, A. *Multiple View Geometry in Computer Vision* 2 edn. (Cambridge University Press, 2006)

[29] Fischler, M. A. & Bolles, R. C. Random sample consensus: A paradigm for model fitting with applications to image analysis and automated cartography. *Communications of the ACM* **24**, 381–395. 10.1145/358669.358692 (1981).

[30] Mount, J. & Milford, M. 2D visual place recognition for domestic service robots at night. In *2016 IEEE International Conference on Robotics and Automation (ICRA)*, 4822–4829. 10.1109/ICRA.2016.7487686 (IEEE, Stockholm, Sweden, 2016).

[31] Horst, M. & Möller, R. Visual place recognition for autonomous mobile robots. *Robotics* **6**, 9. 10.3390/robotics6020009 (2017).

[32] Berganski, C., Hoffmann, A. & Möller, R. Tilt correction of panoramic images for a holistic visual homing method with planar-motion assumption. *Robotics ***12**. 10.3390/robotics12010020 (2023).

[33] Scaramuzza, D., Martinelli, A. & Siegwart, R. A toolbox for easily calibrating omnidirectional cameras. In *2006 IEEE/RSJ International Conference on Intelligent Robots and Systems, IROS 2006, October 9-15, 2006, Beijing, China*, 5695–5701. 10.1109/IROS.2006.282372 (IEEE, 2006).

[34] Urban, S., Leitloff, J. & Hinz, S. Improved wide-angle, fisheye and omnidirectional camera calibration. *ISPRS Journal of Photogrammetry and Remote Sensing* **108**, 72–79 (2015).

[35] Robertson, M. A., Borman, S. & Stevenson, R. L. Dynamic range improvement through multiple exposures. In *Proceedings of the 1999 International Conference on Image Processing, ICIP ’99, Kobe, Japan, October 24-28, 1999*, 159–163. 10.1109/ICIP.1999.817091 (IEEE, 1999).

[36] Schubert, S., Neubert, P., Garg, S., Milford, M. & Fischer, T. Visual place recognition: A tutorial. *IEEE Robotics & Automation Magazine* **31**, 139–153. 10.1109/MRA.2023.3310859 (2024).

[37] Zhang, X., Wang, L. & Su, Y. Visual place recognition: A survey from deep learning perspective. *Pattern Recognition* **113**, 107760. 10.1016/j.patcog.2020.107760 (2021).

[38] Sokolova, M. & Lapalme, G. A systematic analysis of performance measures for classification tasks. *Information processing & management* **45**, 427–437 (2009).

[39] Lowry, S. et al. Visual place recognition: A survey. *IEEE Transactions on Robotics* **32**, 1–19. 10.1109/TRO.2015.2496823 (2016).

[40] Simonyan, K. & Zisserman, A. Very deep convolutional networks for large-scale image recognition. arXiv preprint arXiv:1409.1556 (2014).

[41] He, K., Zhang, X., Ren, S. & Sun, J. Deep residual learning for image recognition. In *Proceedings of the IEEE conference on computer vision and pattern recognition*, 770–778 (2016).

[42] Radenovic, F., Tolias, G. & Chum, O. Fine-tuning CNN image retrieval with no human annotation. *IEEE Transactions on Pattern Analysis and Machine Intelligence* **41**, 1655–1668. 10.1109/TPAMI.2018.2846566 (2019). 29994246 10.1109/TPAMI.2018.2846566

[43] Wang, T.-H. et al. Omnidirectional CNN for visual place recognition and navigation. In *2018 IEEE International Conference on Robotics and Automation (ICRA)*, 2341–2348 (IEEE, 2018).

[44] Payen De La Garanderie, G., Atapour Abarghouei, A. & Breckon, T. P. Eliminating the blind spot: Adapting 3D object detection and monocular depth estimation to 360 panoramic imagery. In Vol. 11217 (eds Ferrari, V., Hebert, M., Sminchisescu, C. & Weiss, Y.) *Computer Vision – ECCV 2018*, 812–830, 10.1007/978-3-030-01261-8_48 (Springer International Publishing, Cham, 2018).

[45] Wang, H. et al. CosFace: Large margin cosine loss for deep face recognition. In *2018 IEEE/CVF Conference on Computer Vision and Pattern Recognition*, 5265–5274. 10.1109/CVPR.2018.00552 (IEEE, Salt Lake City, UT, 2018).

[46] Labrosse, F. The visual compass: Performance and limitations of an appearance-based method. *Journal of Field Robotics* **23**, 913–941 (2006).

[47] Möller, R. A SIMD implementation of the minwarping method for local visual homing. *Bielefeld University, Faculty of Technology, Computer Engineering Group, Tech. Rep.,* (2016).

[48] Möller, R., Horst, M. & Fleer, D. Illumination tolerance for visual navigation with the holistic min-warping method. *Robotics* **3**, 22–67 (2014).

[49] Glorot, X. & Bengio, Y. Understanding the difficulty of training deep feedforward neural networks. In (eds Teh, Y. W. & Titterington, D. M.) *Proceedings of the Thirteenth International Conference on Artificial Intelligence and Statistics, AISTATS 2010, Chia Laguna Resort, Sardinia, Italy, May 13-15, 2010*, vol. 9 of *JMLR Proceedings*, 249–256 (JMLR.org, 2010).

[50] Ioffe, S. & Szegedy, C. Batch normalization: Accelerating deep network training by reducing internal covariate shift. In (eds. Bach, F. R. & Blei, D. M.) *Proceedings of the 32nd International Conference on Machine Learning, ICML 2015, Lille, France, 6-11 July 2015*, vol. 37 of *JMLR Workshop and Conference Proceedings*, 448–456 (JMLR.org, 2015).

[51] Abadi, M. et al. TensorFlow: Large-scale machine learning on heterogeneous systems (2015). Software available from tensorflow.org.

[52] Schroff, F., Kalenichenko, D. & Philbin, J. FaceNet: A unified embedding for face recognition and clustering. In *Proceedings of the IEEE conference on computer vision and pattern recognition*, 815–823 (2015).

[53] Wang, J. et al. Learning fine-grained image similarity with deep ranking. In *Proceedings of the IEEE conference on computer vision and pattern recognition*, 1386–1393 (2014).

[54] DeTone, D., Malisiewicz, T. & Rabinovich, A. SuperPoint: Self-supervised interest point detection and description. In *2018 IEEE/CVF Conference on Computer Vision and Pattern Recognition Workshops (CVPRW)*, 337–33712. 10.1109/CVPRW.2018.00060 (IEEE, Salt Lake City, UT, USA, 2018).

[55] Lindenberger, P., Sarlin, P. & Pollefeys, M. Lightglue: Local feature matching at light speed. In *IEEE/CVF International Conference on Computer Vision, ICCV 2023, Paris, France, October 1-6, 2023*, 17581–17592. 10.1109/ICCV51070.2023.01616 (IEEE, 2023).

[56] Gava, C. et al. SphereGlue: Learning keypoint matching on high resolution spherical images. In *2023 IEEE/CVF Conference on Computer Vision and Pattern Recognition Workshops (CVPRW)*, 6134–6144. 10.1109/CVPRW59228.2023.00653 (IEEE, Vancouver, BC, Canada, 2023).

[57] Nistér, D. An efficient solution to the five-point relative pose problem. *IEEE transactions on pattern analysis and machine intelligence* **26**, 756–770 (2004). 18579936 10.1109/TPAMI.2004.17

[58] Kneip, L. & Furgale, P. T. OpenGV: A unified and generalized approach to real-time calibrated geometric vision. In *2014 IEEE International Conference on Robotics and Automation, ICRA 2014, Hong Kong, China, May 31 - June 7, 2014*, 1–8. 10.1109/ICRA.2014.6906582 (IEEE, 2014).

[59] Differt, D. Real-time rotational image registration. In *2017 18th International Conference on Advanced Robotics (ICAR)*, 1–6. 10.1109/ICAR.2017.8023488 (2017).

