# Supplementary material for: Visual place recognition with panoramic images using hybrid neural network models
Source: Sci Rep. 2026 Jan 8;16:1293. doi: 10.1038/s41598-025-34473-7 (PMC12791132; doi:10.1038/s41598-025-34473-7)
Supplement: Supplementary file 1 — Supplementary Information. [file 41598_2025_34473_MOESM1_ESM.pdf]

Visual Place Recognition with Panoramic Images using Hybrid Neural Network Models – Supplementary Information

Lars Offermann[1,*]

[1]Bielefeld University, Faculty of Technology, Bielefeld, 33615, Germany
[*]loffermann@techfak.uni-bielefeld.de

S1 Maps of Settings

As a reference of the scale of the investigated environments, we provide the overviews in Figure S1, Figure S2, and Figure S3. Measurements of the room dimensions, the location of the area gantry, and the positions of selected furniture pieces were taken using a Leica TS16 3” R500 total station (using single reflectorless measurements). We used a Bosch GLM 40 laser measurement device to validate the lengths of furniture pieces and to get offsets of the capture area from the gantry structure.

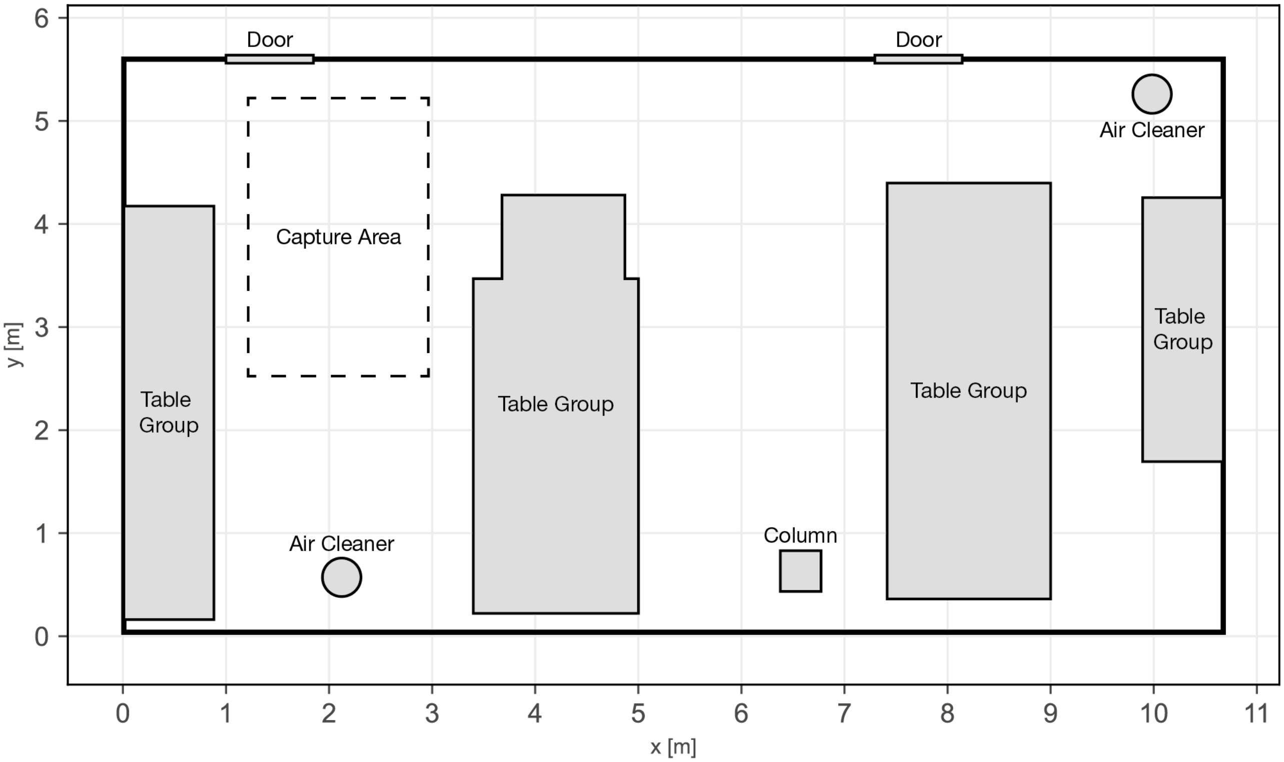

Figure S1. Schematic of placement of the capture area and selected furniture for the setting Computer Lab II.

S2 Error Analysis of Area Gantry Positioning

To be able to use the grid-based image datasets with a spacing as fine as 5 cm for evaluation, we need to ensure that the repeatability and positional accuracy of the area gantry is sufficient. To this end, we place the area gantry in an environment with strictly controlled and even illumination. Then, we collect a range of images by driving the gantry close to the corners of the capture area, which corresponds to the (x, y) positions (0.0 m, 0.0 m), (1.7 m, 0.0 m), (1.7 m, 2.6 m), and (0.0 m, 2.6 m). A single *run* consists of 10 repetitions, after which we return the trolley to the charging position. After each run, we manually move the trolley to ensure contact with charging pads. We record 5 runs. Thus, 50 images are taken at every denoted position. Images are processed as described in Section 3 of the main work, including mapping images to equirectangular of size 99×384 px with three color channels. For each of the selected corners, we calculate the pixel-wise mean squared error between every image. Results are shown in Figure S4.

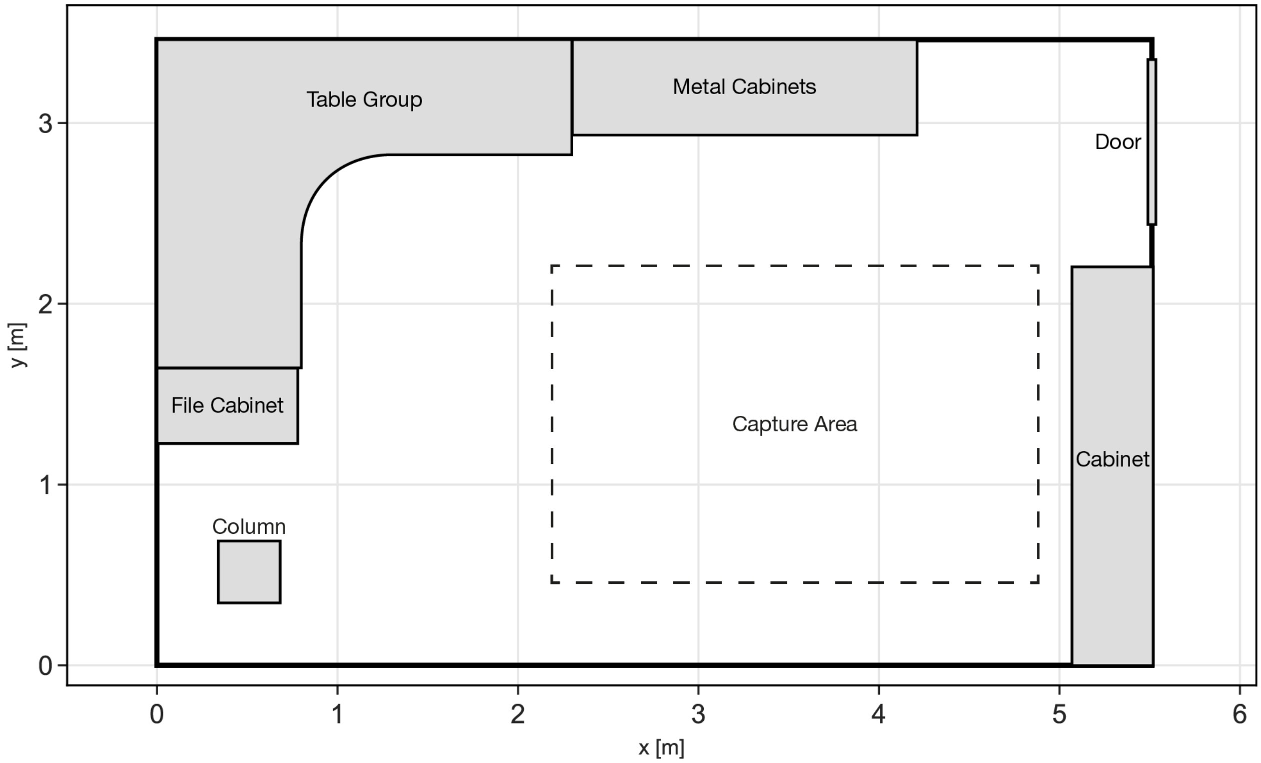

Figure S2. Schematic of placement of the capture area and selected furniture for the setting Gantry Lab.

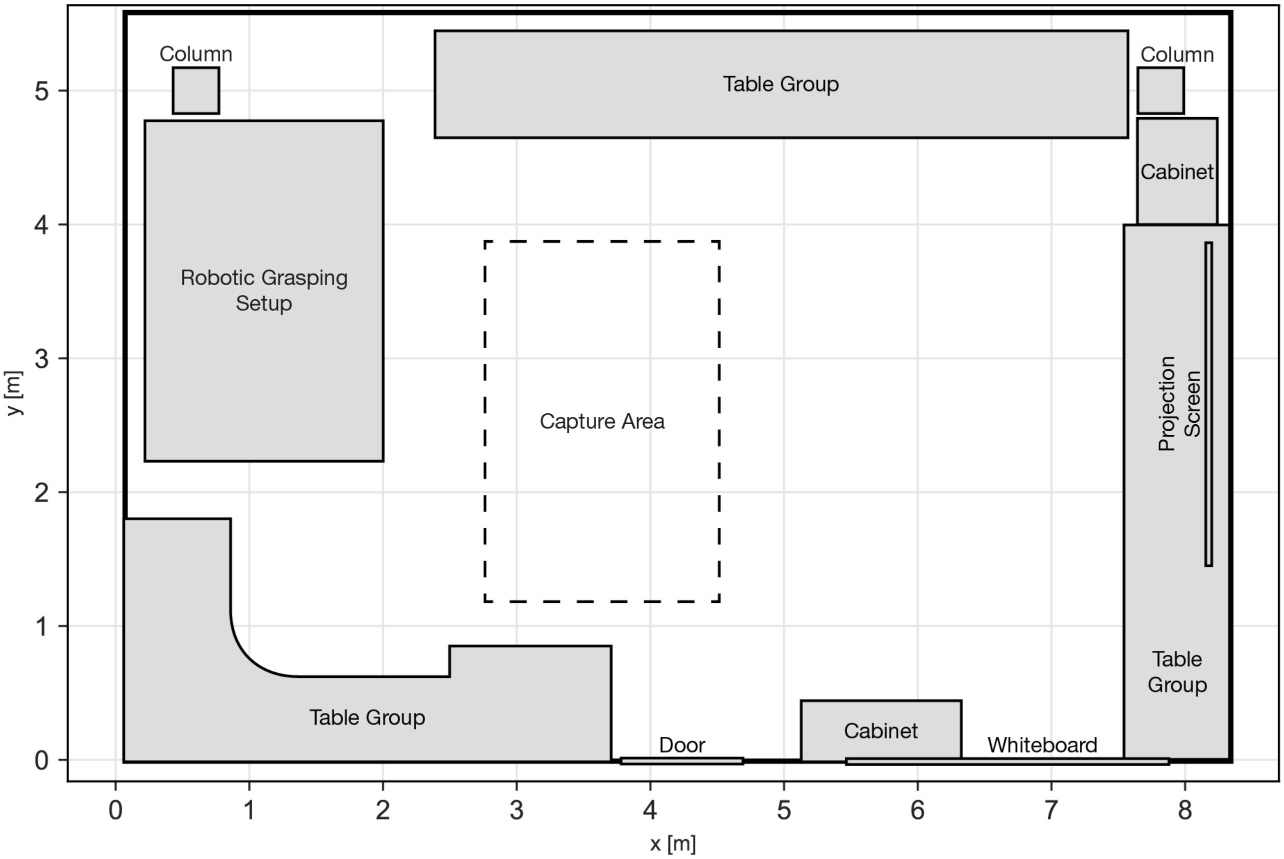

Figure S3. Schematic of placement of the capture area and selected furniture for the setting Robotics Lab II.

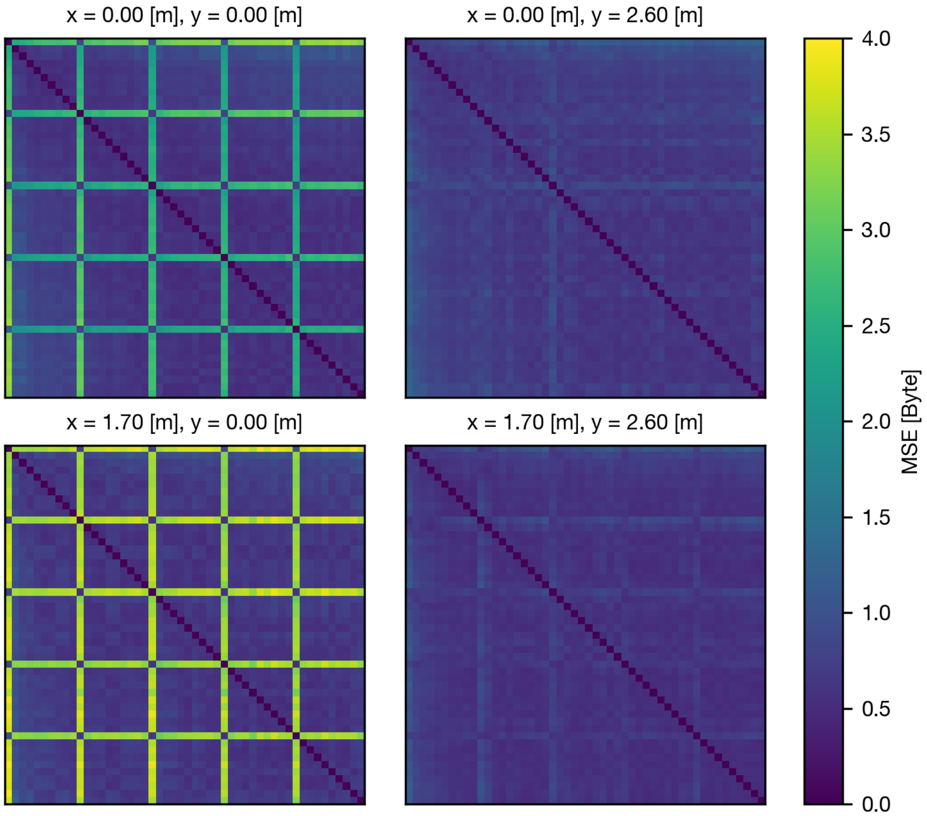

Figure S4. Mean squared error (MSE) between images in an illumination-controlled environment near the corners of the capture area of the gantry.

The first visit to corners close to the x-axis of the gantry (i.e., for y = 0.0 m) exhibit particularly high errors. This is likely caused by manually moving the trolley when establishing contact with the charging pads. Successive visits of the same location within the same and across runs show lower errors, as do any visits to corners for positions at y = 2.6 m.

To visualize the impact of the MSE on image similarity, we show the change between two images by marking pixels with red color in correspondence to the magnitude of the error. To this end, we first compute the pixel-wise squared error, take the average over color channels, then divide by the largest entry. The result is then used as the red and alpha channels of a transparent overlay that is combined with the first image in the pair using alpha compositing. The visualization for the largest error in Figure S4 (at x = 1.7 m, y = 0.0 m) is shown in Figure S5. For reference, we show the error of an image pair that was recorded 5 cm apart in the same environment in Figure S6.

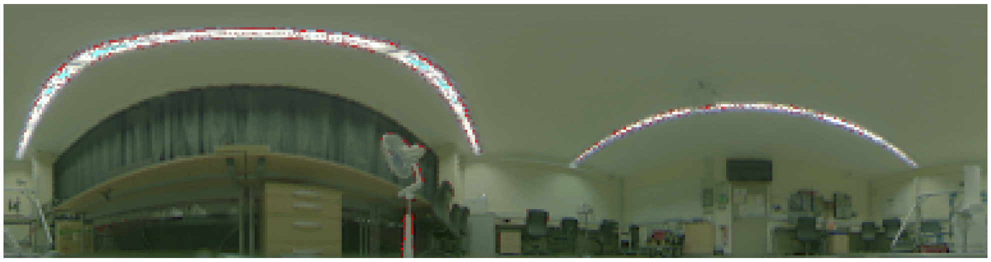

Figure S5. Pixel-wise error within the image pair with the largest recorded error during evaluation of the positioning control of the area gantry, averaged over color channels and overlayed onto the first image in the pair. The greater the intensity of the red color, the larger the pixel-wise error.

The image pair taken at the same position exhibits 1 px-wide regions of errors along parts of the ceiling lights and around the fan assembly, which is one of the closest objects to the camera. Other regions in the images are visually indistinguishable.

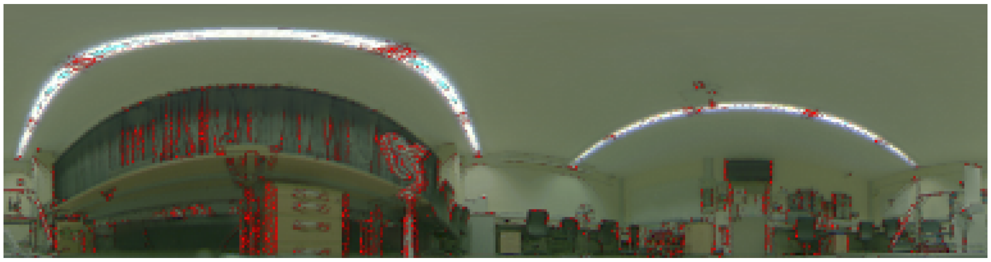

Figure S6. Pixel-wise error for an image pair with a 5 cm distance along the x-axis, with the same processing as Figure S5.

In comparison, the image pair recorded with a 5 cm spacing shows noticeably larger visual changes, especially around the edges of objects. These changes are well distributed throughout the image and become more prominent for objects close to the camera (e.g. the curtains, the file cabinet, and the fan).

Based on this comparison, we determine the remaining error of the area gantry's positioning system small enough to allow evaluation of the VPR algorithms in this work.
